# Life events and their subjective appraisal by children and adolescents in clinical high-risk states of psychosis: a cross-sectional comparison with inpatients with non-psychotic disorders and community subjects

**DOI:** 10.1007/s00787-025-02949-6

**Published:** 2026-02-20

**Authors:** Celina Kullmann, Chantal Michel, Petra Walger, Maurizia Franscini, Nina Traber-Walker, Benno G. Schimmelmann, Rahel Flückiger, Volker Reissner, Frauke Schultze-Lutter

**Affiliations:** 1 Department of Psychiatry and Psychotherapy, Medical Faculty and University Hospital Düsseldorf, Heinrich-Heine-University Düsseldorf , Bergische Landstraße 2, Düsseldorf, 40629 Germany; 2 University Hospital of Child and Adolescent Psychiatry and Psychotherapy, University of Bern, Bern, Switzerland; 3 Department of Child and Adolescent Psychiatry and Psychotherapy, University of Zurich, Zurich, Switzerland; 4 Department of Child and Adolescent Psychiatry, Psychotherapy and Psychosomatics, University of Duisburg-Essen, Essen, Germany; 5 Department of Child and Adolescent Psychiatry, Psychosomatics and Psychotherapy, LVR Clinic Düsseldorf, Düsseldorf, Germany; 6 Department of Psychology, Faculty of Psychology, Airlangga University, Surabaya, Indonesia

**Keywords:** Clinical high-risk for psychosis, Life events, Subjective burden, Subjective appraisal, Negative symptoms, Children and adolescents

## Abstract

While childhood trauma and neglect are generally found to be increased in clinical high-risk of psychosis (CHR-P), findings on life events (LEs) are so far inconclusive, with some indication of a stress sensitization effect in CHR-P, leading to generally higher stress appraisals of LEs, and a LE-reducing effect of negative symptoms. Thus, we studied 50 LEs and their appraisals as assessed with the Munich Life Event Scale in CHR-P patients according to ultra-high risk and basic symptom criteria and age- and sex-matched clinical inpatient (ClinS) and healthy controls (GPS; each *n* = 112, age 8–17 yrs., 57.1% female). LEs, particularly those of interpersonal nature, were increased in CHR-P compared to GPS but not ClinS, and their number and presence was positively related to subjective negative symptoms. Furthermore, appraisals rarely and only unsystematically differed between groups but correlated significantly in most cases with a more negative appraisal being related to a higher stress appraisal. Thus, there was no indication of a significant stress sensitization effect in CHR-P. Rather, the increased number of LEs, in particular interpersonal ones, likely increases the overall load of stress in terms of a stress accumulation and decreases the size of the supportive social network as well as increases negative symptoms. Thus, early interventions in CHR-P should also include improving strategies to deal with LEs, particularly those of interpersonal nature, to improve resilience and reduce burden of non-normative LEs.

**Supplementary Information:**

The online version contains supplementary material available at 10.1007/s00787-025-02949-6.

## Introduction

Psychotic disorders mostly first manifest around the transition from the second to the third decade of life [1]. However, first prodromal symptoms frequently start already in late childhood and adolescence [2, 3], leading to significant burden in this young age group [4]. Thus, an indicated prevention of psychosis in children and adolescents at clinical high-risk for psychosis (CHR-P [5]; henceforth used as an umbrella term) by ultra-high risk (UHR) and/or basic symptom criteria has been considered an urgent need [6, 7]. As a neurodevelopmental disorder of complex multifactorial aetiology, in psychoses, genetic-biological and psychosocial-environmental factors that commonly explain only little variance interact at different levels [8, 9]. Thereby, environmental factors mainly act as a ‘second hit’ on an already vulnerable brain, leading to more or less specific symptoms before first frank psychotic symptoms finally develop [10, 11]. Of the environmental factors, childhood adversities, i.e., neglect and abuse, have received much attention [12–15]; and a reduction of the incidence of psychosis by 33% was suggested could childhood adversities be eliminated from the population [13].

Contrary to childhood adversities, life events (LEs) are considered as part of ‘normal’ life and include both negative and positive changes or situations relating to education and occupation, social relationships, leisure activities, living conditions and health [16, 17]. They can occur as age-related normative LEs with a high probability to occur at a certain age, such as entering or finishing school, and non-normative LEs with a lower probability and little relation to age, such as divorce or moving house [17]. LEs have received less attention in their association to psychosis and CHR-P states, with particular neglect of positive LEs and those not stressful, critical or life-changing by definition [18]. Despite some indication of positive associations of LEs with psychosis, with an increase of LEs in the period leading up to psychosis onset [16], for CHR-P and self-reported psychotic-like experiences (PLEs), results so far are inconclusive and predominately generated on adult or mixed-age samples [19–27]. Some studies indicated a lower number of LEs in UHR or other assumed psychosis-risk samples compared to healthy samples, which was explained as an effect of increasing negative symptoms (NS) of psychosis [14, 19, 24, 25]. Conversely, consistent with higher numbers of childhood adversities [25], recent threatening or negative LEs were also reported as more frequent in UHR patients than in healthy controls and positively associated with PLE severity and NS [21, 26, 27]. Furthermore, differential effects of LEs on UHR states, i.e. negative effects of negative LEs and positive of positive LEs, were reported in a small Mexican adult UHR sample [23], highlighting the importance to consider the entire range of LEs. Moreover, several studies on mainly adult samples with UHR or PLEs [20, 25–27] indicated a major role of subjective stress appraisal of LEs; consistent with a stress-sensitization effect in the development of psychoses – and other mental disorders [29].

To shed more light on the role of a comprehensive range of LEs in CHR-P states in minors, we compared LEs in age- and sex-matched samples of 8- to 17-year-old CHR patients, inpatient controls with non-psychotic disorders (ClinS) and general population control subjects (GPS). In doing so, we paid special attention to the type, number, evaluation (positive or negative), and subjective stress appraisal of the LEs. From earlier studies [20, 25, 28, 30], it was assumed that stress appraisal would best distinguish between CHR-P and control subjects, especially GPS, with CHR-P patients being more stressed by LEs. Furthermore, due to the possible LE-reducing effect of NS [14, 19, 24], we explored the potential interaction of NS severity and presence of LEs, expecting a negative association in CHR-P.

## Methods

### Sample

The sample of 8- to 17-year-olds was recruited as part of the ‘Bi-national Evaluation of At-Risk Symptoms in children and adolescents’ (BEARS-Kid) study [31, 32] at the child and adolescent psychiatric departments of the Universities of Bern (Switzerland), Zurich (Switzerland), and Cologne (Germany). Participants were excluded when they had a psychosis, an IQ ≤ 70, a disturbance due to a general medical condition or substance use, or insufficient German language skills. CHR-P had to meet at least any UHR or basic symptom criteria (sText 1) and were recruited from the local outpatient early detection services. ClinS had to present with any main diagnoses associated with an increased likelihood of developing psychosis in adulthood [33] (sText 1), and were excluded when they had received any antipsychotic medication or were clinically suspected to develop psychosis. GPS were drawn as a random sample from the population register of the greater city of Bern. When ClinS or GPS reported any CHR-P criteria, they were assigned to the CHR-P group (sText 1).

The study was approved by the ethical committees of the University of Bern (No. 174/10), the University of Zurich (No. 2010 − 0415/3), and the University of Cologne (No. 11–071). All participants, and their legal guardians, provided informed written consent prior to study enrolment.

Due to the age-relation of normative LEs [17], subjects of the three groups were matched for age and sex, resulting 112 matched three-pairs (*N* = 336; sText 1), with insignificantly more three-pairs being female (*N* = 64, 57.1%; χ²(1) = 2.29, *p* =.157). Groups did not differ significantly in highest school education, current school class and school completers but ClinS had the most mental disorders and the lowest functioning, closely followed by CHR-P, whose NS were most severe (Table 1). GPS were significantly more likely to be Swiss nationals due to their sole recruitment in Bern. (Table 1).

**Table 1:** Sociodemographic characteristics of the sample (*N* = 336 with each *n* = 112)

	Clinical high risk for psychosis	Clinical controls	Community controls	Statistics
**Sex**, male, %	42.9%	42.9%	42.9%	χ²(2) = 0.000, *p* = 1.0
**Age** (years), mean ± SD	14.9 ± 2.1	14.9 ± 2.1	14.9 ± 2.1	F(2) = 0.003, *p* =.997
**Nationality**, % Swiss German other	58.9% 29.5% 11.6% ⇒	48.2% 47.3% ⇑ 4.5% ⇒	80.4% ⇑ 2.7% ⇒ 17.0% ⇑	χ²(4) = 60.314, *p* <.001
**School (graduation) level** ^a^, % ISCED 1 ISCED 2 ISCED 3	14.3% 55.4% 30.4%	17.0% 50.0% 33.0%	15.2% 58.9% 26.8%	χ²(4) = 1.690, *p* =.792
**Completed school**, %	15.2%	7.1%	10.7%	χ²(2) = 3.705, *p* =.157
**Any current mental disorder** (excl. specific phobia), %	72.3%	100.0% ⇑	6.3% ⇒	χ²(2) = 215.707, *p* <.001
**Number of current disorders**, mean ± SD, median	1.3 ± 1.0, 1	1.5 ± 0.6, 1	0.2 ± 0.4, 0	H(2) = 162.170, *p* <.001; CHR-P = ClinS > GPS
**Functional level** ^b^, mean ± SD, median	61.6 ± 12.8, 60	59.8 ± 11.2, 60	84.4 ± 8.6, 88	H(2) = 171.092, *p* <.001; CHR-P = ClinS < GPS
**Negative Symptoms**, mean ± SD, median subjective (Adynamia) ^c^ attenuated (SIPS-N) ^d^	1.6 ± 1.2, 1.5 1.6 ± 1.0, 1.5	0.8 ± 0.7, 0.7 0.9 ± 0.8, 0.9	0.1 ± 0.2, 0.0 0.2 ± 0.4, 0.0	H(2) = 146.311, *p* <.001; CHR-P > ClinS > GPS H(2) = 156.288, *p* <.001; CHR-P > ClinS > GPS

⇑ 1.96 < standardized residual of cell<−1.96⇒

^a^ International Standard Classification of Education [34] of current school level or highest school graduation, if school had already been completed,

^b^ assessed with the Social and Occupational Functioning Assessment Scale (SOFAS [35]) with 0 = lowest to 100 = highest functioning

^c^ assessed with the SPI-CY (Schizophrenia Proneness Instrument, Child and Youth version [38])

^d^ assessed with the SIPS (Structured Interview for Psychosis Risk Syndromes [39])

### Assessments

LEs were assessed using an age-adapted version of the Münchner Ereignisliste (MEL [36, 37]). The MEL assesses 74 negative and positive, acute and chronic (i.e., present for at least three months) LEs in 12 domains, whereby each domain except one is supplemented by an open question about any further unspecified LEs, resulting in 85 items (sTable 1). LEs were rated for their occurrence within the past five years. Furthermore, most recent single LEs and most troublesome chronic LEs were rated on five-point Likert scales for the subjective appraisal (‘very positive’ to ‘very negative’) and burden (‘not stressful at all’ to ‘extremely stressful’) at the time of their occurrence. Chronic life events were additionally rated for their duration in months. Good test-retest-reliability was reported for the original MEL that can assess LEs over several years [37].

CHR-P criteria, and subjective and attenuated NS were assessed by the Schizophrenia Proneness Instrument, Child & Youth version (SPI-CY [38]) and the Structured Interview of Psychosis-Risk Syndromes (SIPS [39]). In the SPI-CY, the dimension ‘Adynamia’ constitutes of 17 basic symptoms that mainly phenomenologically resemble observable NS but are primarily subjective and self-reported as deviations from the person’s ‘normal’ self, such as reduced energy, impaired stress tolerance, decreased emotional responsiveness, and concentration disturbances. In the SIPS, ratings of the six items of the Negative Symptoms section are primarily based on observable aberrations; these include social anhedonia/withdrawal, avolition, affective flattening, ideational richness, and impairments in occupational functioning. While both scales rate symptoms on a 7-point Likert scale, rating of subjective basic symptoms is based on their frequency, while the syndromal rating of attenuated NS is based on severity defined by anchor points [38, 39]. sText 2 gives further details.

DSM-IV axis-I mental disorders [35] were assessed using the Mini-International Neuropsychiatric Interview for Children and Adolescents (M.I.N.I. KID [40]).

Interviews were conducted by trained clinical psychologist. Children of age 8–12 years were always interviewed in the presence of a parent who was asked to contribute additional information. Adolescents could decide individually about the presence of a parent; those over 14 years-of-age mostly choose to be interviewed alone.

### Data analyses

Using SPSS, version 29, throughout, group comparisons of categorical, and ordinal and non-normally distributed ratio data were calculated using χ²-tests with |standardized residuals|>1.96 indicating significantly deviant cell frequencies, and Kruskal-Wallis-H tests with, in case of *p <*.150, post-hoc Mann-Whitney-U tests (p_adjust_<0.0167). Correlations were calculated using Spearman’s rho.

Stepwise multinomial regression analyses (forward; CHR-P as reference group) were performed to detect the most influential LEs and their potential interaction with the severity of the sum score of subjective and attenuated NS, i.e., the Adynamia and Negative Symptoms sections, respectively.

## Results

### Frequency of LEs

Of the 85 LEs initially surveyed, the 11 open questions were not analyzed due to their diversity and, consequently, non-comparability. Furthermore, 24 LEs, including the entire domain ‘pregnancy/children’, were never reported and, thus, not analyzed (sTable 1). Consequently, 50 LEs (Table 2) entered further analyses.

**Table 2:** Frequency of past-year-LEs (each group *n* = 112)

LE No.	LE (short form)	Frequency of any past-year-LEs % (⇑ 1.96 < standardized residual<−1.96 ⇒)			
		CHR-*P*	ClinS	GPS	χ²(2), *p*
1	School-started	16.1%	16.1%	10.7%	1.750, 0.417
2	School-completed	6.3%	2.7%	9.8%	4.876, 0.087
3	School-failed	2.7%	0.9%	1.8%	1.018, 0.601
4	School-partner completed	0.9%	0.0%	0.0%	2.006, 0.367
8	Work^a^-resigned	0.0%	0.0%	0.0%	n.a.
9	Work^a^-new job	0.9%	0.0%	0.9%	1.006, 0.605
14	Work^a^-new tasks	1.8%	2.7%	2.7%	0.256, 0.880
15	Work^a^-changed conditions	8.9%	6.3%	5.4%	1.214, 0.545
16	Work^a^-unemployed	0.9%	0.0%	0.0%	2.006, 0.367
17	Work^a^-sick-leave	7.1%	18.8% ⇑	0.0% ⇒	25.437, < 0.001
19	Work^a^-disputes	23.2% ⇑	17.0%	5.4% ⇒	14.286, < 0.001
20	Work^a^-overload	25.0% ⇑	17.9%	2.7% ⇒	22.608, < 0.001
21	Work^a^-satisfying	11.6%	6.3%	6.3%	2.900, 0.235
23	Main earner^b^-promotion	2.7%	4.5%	0.0%	4.866, 0.088
24	Main earner^b^-dismissal	4.5% ⇑	0.9%	0.0%	7.127, 0.028
25	Main earner^b^-unemployed	7.1%	3.6%	0.9%	5.921, 0.052
27	Love-started	17.9% ⇑	8.9%	5.4%	9.707, 0.008
28	Love-move in together	0.0%	0.0%	0.0%	n.a.
29	Love- ended	15.2% ⇑	6.3%	4.5%	9.360, 0.009
32	Love-local distance	0.0%	0.0%	0.9%	2.006, 0.367
36	Love-no partner	13.4% ⇑	5.4%	4.5%	7.587, 0.023
37	Love-no sex	0.9%	0.0%	0.0%	2.006, 0.367
38	Love-disputes	1.8%	2.7%	0.0%	2.842, 0.241
39	Love-satisfying	2.7%	4.5%	2.7%	0.752, 0.687
51	Parents-moved out	3.6%	0.9%	0.0%	5.279, 0.071
52	Parents-dispute	24.1% ⇑	17.0%	0.9% ⇒	26.320, < 0.001
53	Parents-satisfying	12.5%	15.2%	8.0%	2.781, 0.249
54	Parents-moved back in	0.9%	0.0%	1.8%	2.018, 0.365
55	Relatives-dispute	8.0%	7.1%	0.0% ⇒	9.046, 0.011
57	Friendship-started	43.8%	50.9%	28.6% ⇒	12.026, 0.002
58	Friendship-ended	15.2%	19.6%	13.4%	1.721, 0.423
59	Friendship-none	7.1%	12.5% ⇑	0.0% ⇒	14.397, < 0.001
60	Leisure-restrictions	12.5%	21.4% ⇑	4.5% ⇒	14.454, < 0.001
61	Leisure-increases	8.9%	10.7%	5.4%	2.182, 0.336
62	Friendship-stressful	5.4%	2.7%	3.6%	1.120, 0.571
63	Friendship-satisfying	15.2%	10.7%	6.3%	4.667, 0.097
67	Death-parent	0.9%	0.9%	0.0%	1.006, 0.605
68	Death-friend/relative	8.9%	10.7%	12.5%	0.747, 0.688
69	Housing-relocation	7.1%	8.0%	8.9%	0.242, 0.886
71	Housing-poor condition	0.0%	0.9%	0.0%	2.006, 0.367
73	Income-increased	1.8%	0.9%	0.0%	2.018, 0.365
74	Income-deteriorated	1.8%	0.0%	0.0%	4.024, 0.134
75	Income-too low	0.0%	0.0%	0.9%	2.006, 0.367
77	Court-sentencing	0.0%	0.9%	0.0%	2.006, 0.367
78	Court-proceedings	5.4%	3.6%	7.1%	1.409, 0.494
80	Health-hospital^c^	12.5% ⇑	4.5%	0.0% ⇒	16.847, < 0.001
81	Relative-hospital	15.2%	14.3%	3.6% ⇒	9.537, 0.008
82	Health-treatment	35.7% ⇑	40.2% ⇑	0.9% ⇒	54.416, < 0.001
83	Relative-treatment	14.3%	9.8%	4.5%	6.288, 0.043
84	Health-stressed	0.9%	0.0%	0.0%	2.006, 0.367

*CHR-P *clinical high-risk for psychosis, *ClinS* inpatient controls, *GPS* general population/community controls

^a^ “Work” includes any vocational trainings or employments undertaken after completing full-time compulsory schooling that in Switzerland and Germany ends at the age of 15/16 years

^b^ “Main earner” refers to the person who is the primary breadwinner of the participant’s family and provides the main financial support to the participant, usually a parent

^c^ excluding current hospital stay

Overall, 97% of participants had reported any LEs in the past five years (henceforth: five-year-LEs), with no significant difference between groups (χ²(2) = 1.443, *p* =.486). For the past year, however, significantly less GPS (63.4%) than CHR-P (89.3%) and ClinS (93.8%) had reported any LEs that had newly occurred or was still ongoing (henceforth: past-year-LEs; χ²(2) = 41.026, *p* <.001; Cramer’s V = 0.349). Furthermore, both CHR-P (4.3 ± 3.2, Mdn = 4.0) and ClinS (3.8 ± 2.6, Mdn = 3.5) reported a higher number of different past-year-LEs compared to GPS (1.8 ± 2.1, Mdn = 1.0; H(2) = 58.492, *p* <.001).

LEs relating to education, social contacts/leisure activities, and health/illness were both most and similarly frequent in all groups (Table 2; sTable 2). GPS reported significantly fewer negatively perceived LEs than CHR-P and ClinS in the domains occupation, social contacts/leisure activities, parents/family and health/illness (Table 2, sTable 2) but the most school completions in the previous five years (LE-2).

The regression models of five-year-LEs and past-year-LEs explained around 50% of the variance, whereby the past-year-LEs-model had better fit (sTables 3–4). Mostly, both models indicated a higher number of LEs in CHR-P, in particular of the domains occupation, parents/family and health/illness.

### Subjective appraisal (positive vs. negative) and burden (not stressful vs. stressful) of LEs

Despite a general trend towards more negative and more stressful appraisals of LEs in the two clinical groups, even for commonly positive LEs, such as a new love (LE-27) or friendship (LE-36), or a satisfying work (LE-21), evaluation scores of LEs rarely differed significantly between groups (sTables 2, 5). Only for three five-year LEs – start of school (LE-1), moving (LE-69) and treatment (LE-82) – and one past-year-LE, no partner (LE-36), a significantly more negative evaluation was accompanied by a significantly more stressful evaluation (sTable 2, sTable5).

Overall, subjective appraisal and burden of past-year-LEs commonly correlated at least moderately (rho ≥ 0.300), with more negative evaluations being correlated to more stressful appraisals (sTable 6). Only in two LEs, new work-tasks (LE-14) and moving out of family home (LE-51), evaluations correlated not at all; in four past-year-LEs, they correlated only to a weak degree: dismissal of main earner (LE-24), new love (LE-27), satisfying relation with parents (LE-53) and new friendship (LE-57) (sTable 6).

### Frequency of LEs and negative symptoms

Both subjective and attenuated NS played a significant role in the regression models (sTables 7, 8, 9, 10, 11, 12, 13 and 14) and were even the only selected variable in models with the sum of any five-year-LEs (sTables 7, 8, 9 and 10). In case of the models of the sum of or any past-year-LEs, attenuated NS and the respective LE-variable both entered the models independently (sTable 11, and 12). Subjective NS were an independent predictor in models of single five-year- and past-year-LEs (sTable 13, and 14).

In models of single five-year- and past-year-LEs including attenuated NS, next to being an independent predictor, these also significantly interacted with treatment (LE-82) and, in the five-year-LEs model, additionally with chronic dispute with parents (LE-52)(sTables 15–16). Thereby, treatment was associated with higher attenuated NS scores in CHR-P but with slightly lower scores in ClinS and GPS (sFigures 1–2). Chronic dispute with parents was related to higher attenuated NS in all groups, and while this was slightly so in both clinical groups, it was extreme in GPS (sFigure 3).

**Fig. 1:**
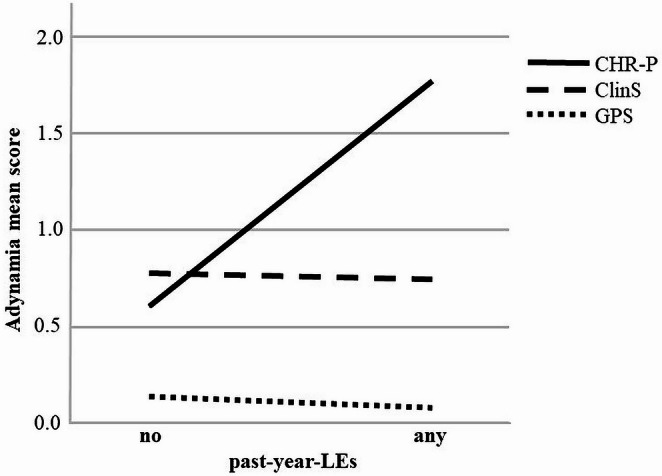
Interaction of subjective negative symptoms (Adynamia) with presence of any past-year-LEs CHR-P: clinical high-risk for psychosis; ClinS: inpatient controls; GPS: general population/community controls

With regard to past-year-LEs, the models of both their sum or the presence of any one included the summary-variable, Adynamia and the interaction of both (Tables 3 and 4). Presence of any past-year-LEs was associated with higher subjective NS scores in CHR-P but with slightly lower scores in ClinS and GPS (Fig. 1). The sum of past-year LEs was moderately positively correlated with the Adynamia score in CHR-P (rho = 0.319) but only weakly positively correlated in ClinS (rho = 0.088) and GPS (rho = 0.133).

**Table 3:** Stepwise multinomial regression model of sum of past-year-LEs and subjective negative symptoms (Adynamia mean score)

	Clinical High-Risk for Psychosis vs. Community Controls							Clinical High-Risk for Psychosis vs. Clinical Controls						
	β (SE)	SE	Wald (df = 1)	*p*	Exp(β)	95% CI of Exp(β)		β (SE)	SE	Wald (df = 1)	*p*	Exp(β)	95% CI of Exp(β)	
						**lower**	**upper**						**lower**	**upper**
Intercept	2.268	0.361	39.429	<0.001				0.544	0.357	3.326	0.127			
Adynamia	−3.386	0.881	14.783	< 0.001	0.034	0.006	0.190	−0.448	0.283	2.506	0.113	0.639	0.367	1.113
Sum of past-year-LEs	−0.083	0.111	0.564	0.453	0.920	0.741	1.143	0.164	0.089	3.392	0.065	1.178	0.990	1.403
Adynamia*Sum of past-year-LEs	−0.485	0.347	1.953	0.162	0.615	0.312	1.216	−0.135	0.065	4.343	0.037	0.874	0.770	0.992

*SE* standard error, *CI* confidence interval; a: not calculable due to too low frequency in at least one group

R²=0.555 (Nagelkerke), χ²(6) = 228.232, *p* <.001, AIC = 387.327, BIC = 417.864

**Table 4:** Stepwise multinomial regression model of any past-year-LEs and subjective negative symptoms (Adynamia mean score)

	Clinical High-Risk for Psychosis vs. Community Controls							Clinical High-Risk for Psychosis vs. Clinical Controls						
	β (SE)	SE	Wald (df = 1)	*p*	Exp(β)	95% CI of Exp(β)		β (SE)	SE	Wald (df = 1)	*p*	Exp(β)	95% CI of Exp(β)	
						**lower**	**upper**						**lower**	**upper**
Intercept	1.911	0.443	18.620	<0.001				−0.759	0.666	1.299	0.254			
Adynamia	−2.340	0.885	6.987	0.008	0.096	0.017	0.546	0.318	0.652	0.237	0.626	1.374	0.383	4.933
Any past-year-LEs	0.290	0.533	0.296	0.586	1.337	0.470	3.801	2.141	0.714	8.986	0.003	8.510	2.098	34.515
Adynamia*Any past-year-LEs	−3.555	1.270	7.832	0.005	0.029	0.002	0.345	−1.434	0.677	4.480	0.034	0.238	0.063	0.899

*SE* standard error, *CI* confidence interval; a: not calculable due to too low frequency in at least one group

R²=0.566 (Nagelkerke), χ²(6) = 234.839, *p* <.001, AIC = 256.396, BIC = 286.933

## Discussion

This study aimed to shed more light on the frequency and appraisal of LEs in minors at CHR-P, and their interaction with NS. Contrary to our expectations, we found only few and mostly unsystematic differences between CHR-P and the two control groups, especially no indication of a generally significantly higher burden in CHR-P. Compared to GPS, CHR-P reported a similar or higher number of LEs. In doing so, next to the sample-inherent higher number of health-related LEs that CHR-P shared with ClinS, increased numbers of LEs occurred in the interpersonal LE-domains, indicating unstable love relationships, and more conflicts with parents and at work, as well as more experiences of work overload. Furthermore, we found no indication of a LE-reducing effect of NS. On the contrary, especially in CHR-P, the number and presence of any past-year-LEs were positively correlated with the severity of subjective NS as measured by the Adynamia dimension of the SPI-CY.

### CHR-P and interpersonal LEs

More interpersonal LEs in CHR-P compared to healthy controls were also found in two other studies [21, 26] that suggested this being caused by a heightened sensitivity to interpersonal issues driven by attenuated psychotic symptoms, e.g., that paranoid ideas may lead to mistrust in the social environment and, consequently, to increased interpersonal LEs [21, 26]. Alternatively, increased interpersonal LEs may increase stress, which is considered a risk factor for psychosis, and may act as a catalyst for further psychotic experiences [26] or, as proposed in cognitive models, the appraisal of interpersonal events may result in negative schemas, leading to the development of attenuated psychotic symptoms [21]. Longitudinal studies are therefore needed to examine the temporal relationship between LEs and CHR-P symptoms.

Interpersonal problems, especially unstable relationships and conflicts due to emotional instability, are core features of borderline personality disorder (BPD) [35, 41], which has been linked to UHR, but not to basic symptom criteria [42–46]. In a mixed-age UHR sample, 62–78% showed affective instability, anger, impulsivity, and unstable relationships per DSM-IV BPD criteria [42]. Thus, the higher rate of interpersonal problems in our CHR-P sample may reflect emerging borderline traits. As personality disorders were not assessed due to the DSM-IV age threshold, future studies should consider their role. With DSM-5 Section III and ICD-11 now emphasizing personality functioning—particularly interpersonal functioning—as central to early personality pathology [47, 48], examining this domain in CHR-P individuals is especially warranted.

Finally, the high number of interpersonal LEs may indicate an erosion of supportive social networks and/or an impediment in the normal growth in social network size across adolescence [17]. Both interpretations that require further longitudinal studies would be in line with the reported smaller social networks overall, whereby social support deficits were specific to relatives other than parents and friends [49]. Since good social networks can act as an environmental protective factor not only for psychosis but also for suicidality in CHR-P states, social relationships were suggested a promising early intervention target in CHR-P, e.g., by expressed emotion interventions or social skills training [49, 50].

### CHR-P and stress susceptibility

Experiences of chronic work overload that were most frequent in CHR-P – even in the absence of significantly increased occurrence of other work/school-situation-related LEs – may be considered the most direct expression of an increased stress susceptibility of all assessed LEs. Yet, as work overload was commonly reported as stressful, no additional significant group differences in stress appraisal were found, despite descriptively higher stress ratings in the two clinical groups. This was also true for most other LEs for that a significantly higher stress appraisal in CHR-P was extremely rare and unsystematic, and even less among past-year-LEs compared to five-year-LEs. Overall, our results therefore do not support a major role of subjective stress appraisal of LEs in terms of a stress-sensitization effect [20, 25–29]. Accordingly, a recent study using Ecological Momentary Assessment [51] reported no contemporaneous or temporal link between stress on CHR-P symptoms but rather a contemporaneous effect of CHR-P symptoms on stress. Thus, in addition to CHR-P symptoms, the higher number of mainly negative LEs might have a cumulative effect on the overall stress level in terms of a stress accumulation that increasingly rises the activity of the HPA and cortisol level during the time of emerging psychosis [52, 53].

Following developmental models of psychosis, increased negative or stressful LEs can lead to the critical stress threshold being exceeded, especially when protective factors including resilience and adaptive coping strategies fail to buffer against these stressors [8, 54]. Accordingly, UHR patients were more likely to exhibit emotional coping strategies that are mainly used in situations perceived as not changeable, while GPS used more task-orientated coping strategies that are mainly used in situations perceived as changeable [24]. This suggested that in themselves stressful feelings of little control over events by CHR-P might be triggered by experiences of low social support [24, 55]. Therefore, treatment strategies that focus on stress management and improving coping skills may help mitigate the negative effects of accumulating, especially normative and therefore largely unavoidable LEs [24].

### LEs and negative symptoms

Contrary to the assumed LE-reducing effect of NS, leading to lower numbers of LEs in CHR-P [14, 19, 24], in CHR-P, we detected neither fewer LEs nor negative correlations, where NS and LEs interacted. Rather, positive correlations between NS and LEs were commonly the most pronounced in CHR-P compared to other groups; only dispute with parents, was mostly related to NS in GPS. A positive association of NS and stressful LEs in UHR was also reported by See et al. [21], with LEs not contributing to a worsening of symptoms over time. Yet, when cannabis use was also taken into account, LEs were no longer significantly associated with NS [21].

Although NS were significantly less severe in ClinS and GPS compared to CHR-P, a slight negative association of LEs and NS was observed in ClinS and GPS only for treatment and presence of any past-year-LEs, again with a positive association in CHR-P. The interaction with treatment might indicate that observable attenuated NS, including affective symptoms that were reported a major reason for help-seeking in CHR-P [56], played a greater role in the decision to seek help in CHR-P compared to ClinS and GPS. Whereas the interaction with presence of past-year-LEs possibly indicate differences in coping with subjective NS [24] that might result in more LEs in CHR-P. Thus, improving coping strategies for subjective NS, e.g., by cognitive-behavioural interventions, may prevent avoidable non-normative LEs, especially interpersonal ones. Yet, more detailed and longitudinal studies are needed to shed more light on LE-NS interactions in the various groups.

### Strengths and limitations

Next to the strengths of our study – such as the assessment of the whole range of LEs, not only negative or per se stressful ones, the matching of samples to avoid age-related impact of normative LEs, and assessment of appraisals of LEs, some additional limitations need to be addressed. First, results will be impacted by differences in study design, such as assessment of LEs or of stress and stress tolerance – related to a specific LE or assessed as an independent variable [27, 30, 57], sample composition including the unknown but likely different proportion of CHR-P who will actually develop psychosis [52] and analyses [57]. Second, most LE studies were conducted in adult or mixed-age samples with different normative LEs than present in childhood and early adolescence [17]. Furthermore, parental mental health and parenting behavior also play a much stronger role in the interplay of LEs and mental health [58]; thus, these should be included in future studies of LEs in this young age group. This is also true for other so far not mentioned potential moderators and mediators such as: positive and other symptoms [21, 25, 28], comorbidities, particularly affective and anxiety disorders [58, 59], resilience factors [24], treatments (including different types of medication) [60], and biogenetic parameters of stress [26, 30, 53]. These should be studied using methods that allow studying their interplay, such as structural equation analyses [58], and disentangle cause and effect in longitudinal designs.

## Conclusions

Alike childhood trauma and neglect, LEs were increased in CHR-P states. While there was no indication of a significant stress sensitization effect that leads to generally higher stress appraisals of LEs, the increased number of LEs, in particular interpersonal ones, will likely increase the overall load of stress in terms of a stress accumulation and decrease the size of the supportive social network as well as increase NS. Thus, early interventions in CHR-P should also include improving strategies to deal with LEs, particularly those of interpersonal nature, to improve resilience and coping with NS and to reduce burden of non-normative LEs.

## Supplementary Information

Below is the link to the electronic supplementary material.

Supplementary Material 1 (DOCX 316 KB)

## Data Availability

The datasets used in this study are available from the corresponding author upon reasonable request, due to privacy and ethical restrictions.
