# Supplementary material for: Life events and their subjective appraisal by children and adolescents in clinical high-risk states of psychosis: a cross-sectional comparison with inpatients with non-psychotic disorders and community subjects
Source: Eur Child Adolesc Psychiatry. 2026 Feb 20;35(5):1677–87. doi: 10.1007/s00787-025-02949-6 (PMC13272262; doi:10.1007/s00787-025-02949-6)
Supplement: Supplementary file 1 — Supplementary Material 1 (DOCX 316 KB) [file 787_2025_2949_MOESM1_ESM.docx]

**Supplementary Material to**

***Life events and their subjective appraisal by children and adolescents in clinical high-risk states of psychosis: a cross-sectional comparison with inpatients with non-psychotic disorders and community subjects***

**by Kullmann C. et al.**

Contents

[sText 1: Additional information on the recruitment of samples in the Binational Evaluation of At-Risk Symptoms in Children and Adolescents (BEARS-Kid) study 2](#_Toc197524738)

[sTable 1: Overview of life events included in the MEL and their short form used in this paper 5](#_Toc197524739)

[sText 2: Subjective and attenuated negative symptoms assessed with the SPI-CY and SIPS, respectively 7](#_Toc197524740)

[sTable 2: Frequency and subjective appraisals of five-year-LEs (all groups n=112) 9](#_Toc197524741)

[sTable 3: Stepwise multinomial regression model of five-year-LEs with significant frequency differences (see sTable 2) 12](#_Toc197524742)

[sTable 4: Stepwise multinomial regression model of past-year-LEs with significant frequency differences (see Table 2) 12](#_Toc197524743)

[sTable 5: Subjective appraisals of reported past-year-LEs (all groups n=112) 13](#_Toc197524744)

[sTable 6: Correlations of the subjective appraisals of past-year-LEs in the total sample (n=336) 15](#_Toc197524745)

[sTable 7: Stepwise multinomial regression model of sum of five-year-LEs and subjective negative symptoms (Adynamia mean score) 16](#_Toc197524746)

[sTable 8: Stepwise multinomial regression model of any five-year-LEs and subjective negative symptoms (Adynamia mean score) 16](#_Toc197524747)

[sTable 9: Stepwise multinomial regression model of sum of five-year-LEs and attenuated negative symptoms (SIPS-N mean score) 16](#_Toc197524748)

[sTable 10: Stepwise multinomial regression model of any five-year-LEs and attenuated negative symptoms (SIPS-N mean score) 17](#_Toc197524749)

[sTable 11: Stepwise multinomial regression model of any past-year-LEs and subjective negative symptoms (Adynamia mean score) 17](#_Toc197524750)

[sTable 12: Stepwise multinomial regression model of any past-year-LEs and attenuated negative symptoms (SIPS-N mean score) 17](#_Toc197524751)

[sTable 13: Stepwise multinomial regression model of five-year-LEs with significant frequency differences and subjective negative symptoms (Adynamia mean score) 18](#_Toc197524752)

[sTable 14: Stepwise multinomial regression model of past-year-LEs with significant frequency differences and subjective negative symptoms (Adynamia mean score) 18](#_Toc197524753)

[sTable 15: Stepwise multinomial regression model of five-year-LEs with significant frequency differences and attenuated negative symptoms (SIPS-N mean score) 19](#_Toc197524754)

[sTable 16: Stepwise multinomial regression model of past-year-LEs with significant frequency differences and attenuated negative symptoms (SIPS-N mean score) 20](#_Toc197524755)

[sFigure 1: Interaction of treatment (LE-82) within the past five years and attenuated negative symptoms (SIPS-N)……………………………. 21](#_Toc197524756)

[sFigure 2: Interaction of treatment (LE-82) within the past year and attenuated negative symptoms (SIPS-N)…………………………. 21](#_Toc197524757)

[sFigure 3: Interaction of chronic dispute with parents (LE-52) within the past five years and attenuated negative symptoms (SIPS-N) 22](#_Toc197524758)

[sTable 17: STROBE Statement for cohort studies 23](#_Toc197524759)

## sText 1: Additional information on the recruitment of samples in the Binational Evaluation of At-Risk Symptoms in Children and Adolescents (BEARS-Kid) study

For **inclusion in the clinical high-risk of psychosis (CHR-P) group**, any one ultra-high risk [Yung et al., 1998; Phillips et al., 2000] or basic symptom criterion [SchultzeLutter et al., 2015, 2016] had to be fulfilled. The basic symptom criteria alternatively include the Cognitive Disturbances (COGDIS) and the Cognitive-Perceptive basic symptoms (COPER) criterion [Schultze-Lutter & Koch, 2010], the ultra-high risk criteria include the Attenuated Positive Symptom Syndrome (APSS), the Brief Intermittent Psychosis Syndrome (BIPSS) and the Genetic Risk and Functional Deterioration Syndrome (GRDS) [McGlashan et al., 2010]. One-hundred thirty-seven CHR-P participants were recruited in the three early detection services, additional 46 participants of the community and inpatient sample met CHR-P criteria and were registered as members of the **CHR-P sample** that, finally, included **N=183** participants (sFigure A).

Of the **112 CHR-P** participants included in these analyses, five (4.5%) exclusively met GRFD, 41 (36.6%) met basic symptom criteria – two in addition to GRFD, 25 (22.3%) met APSS – three in addition to GRFD, and 41 (36.6%) met the combination of APSS (and BIPSS) and basic symptom criteria.


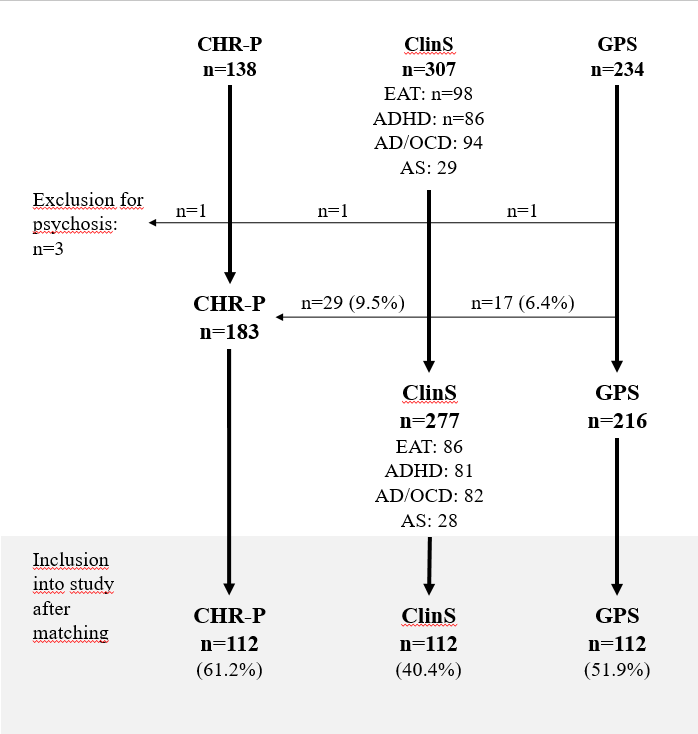
**sFigure A. Recruitment of the analysed sample (N=336).**

CHR-P: clinical high-risk for psychosis;

ClinS: inpatient controls;

GPS: general population/ community controls;

EAT: eating disorders;

ADHD: attention deficit/ hyperactivity disorders;

AD/OCD: anxiety including obstetric-compulsive disorders;

AS: Asperger’s disorder

For **inclusion in the inpatient group (ClinS)**, the main diagnosis according to the 4th edition of the Diagnostic and Statistical Manual of Mental Disorders (DMS-IV [American Psychiatric Association, 1994]) had to be one for which Rubino et al. [2009] had reported at least a 2.5 times increased prevalence of subsequent schizophrenia:

- attention deficit hyperactivity disorder (ADHD) (inattentive, hyperactive and impulsive subtype);
- anxiety disorders (social and severe specific phobia, mainly school phobia);
- obsessive–compulsive disorder; and
- eating disorder (anorexia and bulimia nervosa).

In addition, patients with Asperger’s syndrome were included. Asperger’s syndrome had not been considered by Rubino et al. [2009] but has been recognized explicitly as a developmental disorder with an increased risk of psychotic episodes in young adulthood in DSM-IV. Because co-occurrence of mental disorders is rather the rule than the exception in patients with mental disorders, in clinical as well as in community samples [Noterdaeme et al., 2004; Alonso et al., 2004], we did not use (co-) morbidities with non-psychotic mental disorders as an exclusion criterion in any sample, in order not to limit representativeness. We recruited N=306 initial inpatients: n=97 with eating disorders, n=86 with ADHD, n=94 with anxiety and obsessive–compulsive disorders, and n=29 with Asperger’s syndrome. Despite not being clinically suspected to develop psychosis, n=29 of them met CHR-P criteria and joined the CHR-P group, leaving **ClinS of N=277** (sFigure A).

Of the **112 ClinS** included in these analyses, 19 (17.0%) had a primary diagnosis of ADHD, 25 (22.3%) of anxiety disorder, 13 (11.6%) of obsessive-compulsive disorder, 42 (37.5%) of eating disorder, and 13 (11.6%) of Asperger’s syndrome.

For the **recruitment of a representative general population sample (GPS)**, the Agency for Informatics and Organization of the Canton Bern, Switzerland, randomly drew a sample of N=980 persons (including addresses) stratified for age and sex from the population register of the city of Bern and its urban hinterland, an area comprising approximately 200 000 residents. Subsequently, we searched directories and the Internet for telephone numbers, because the availability of a working telephone number served as an **eligibility criterion** in this group. For n=176 (18.0%) of the register-drawn persons, we could not ascertain a working telephone number, and n=41 (4.2%) persons were drawn twice. First contact with the remaining n=763 (77.9%) persons was established by an information letter, personally addressing each potential participant and his/her legal guardians. Next, we contacted parents and/or their children by telephone, informed them in detail, and asked them to give written informed consent and assent. In children below age 16.0 years, we contacted parents first. Of these, n=233 children and adolescents (30.5%), and their legal guardians agreed to participate. Seventeen of them (7.3%) met CHR-P criteria and joined the CHR-P group, resulting in a final **GPS of N=216** (Figure A). A total of n=353 (46.3% of the contacted sample) contacted persons did not agree to participate, mainly for lack of interest (35.6%) or time (35.9%). Additionally, we had to excluded n=52 persons (6.8%) because they had reached the age of 18 years by the time contact was made (53.9%), had moved away from the greater Bern area (32.7%), or lacked the ability to participate in the study for language or physical health reasons (13.5%). With n=124 persons (16.3%), all attempts (at least 40) to reach them on the telephone remained fruitless.

Thus, according to the standard definitions of the American Association for Public Opinion Research [2016], the contact rate was 82.7%, the cooperation rate was 39.9%, the refusal rate was 49.2%, and the response rate was 32.6%.

**References of sText 1**:

Alonso J, Angermeyer MC, Bernert S, Bruffaerts R, Brugha TS, Bryson H, de Girolamo G, Graaf R, Demyttenaere K, Gasquet I, Haro JM, Katz SJ, Kessler RC, Kovess V, Lépine JP, Ormel J, Polidori G, Russo LJ, Vilagut G, Almansa J, Arbabzadeh-Bouchez S, Autonell J, Bernal M, Buist-Bouwman MA, Codony M, Domingo-Salvany A, Ferrer M, Joo SS, Martínez-Alonso M, Matschinger H, Mazzi F, Morgan Z, Morosini P, Palacín C, Romera B, Taub N, Vollebergh WA (2004) 12-Month comorbidity patterns and associated factors in Europe: results from the European Study of the Epidemiology of Mental Disorders (ESEMeD) project. Acta Psychiatr Scand Suppl:28-37. https://doi.org/10.1111/j.1600-0047.2004.00327.x

American Association for Public Opinion Research (2016). Standard Definitions: Final Dispositions of Case Codes and Outcome Rates for Surveys. 9th edition. AAPOR

American Psychiatric Association (1994). Diagnostic and statistical manual of mental disorders: DSM-IV. Washington, American Psychiatric Association.

McGlashan TH, Walsh B, Woods SW (2010). The psychosis-risk syndrome. Handbook for diagnosis and follow-up. New York: Oxford University

Noterdaeme M, Schlamp D, Linder M, Kischel KH (2004). Analyse der komorbiden psychiatrischen Diagnosen anhand der Basisdokumentation der Kinder- und Jugendpsychiatrie [Analysis of comorbid psychiatric disorders in child and adolescent psychiatry using the standardised basic documentation]. Psychiatr Prax 31 Suppl 1:S126-128. https://doi.org/10.1055/s-2004-828452

Phillips LJ, Yung AR, McGorry PD (2000) Identification of young people at risk of psychosis: validation of Personal Assessment and Crisis Evaluation Clinic intake criteria. Aust N Z J Psychiatry 34 Suppl:S164-169. https://doi.org/10.1080/000486700239

Rubino IA, Frank E, Croce Nanni R, Pozzi D, Lanza di Scalea T, Siracusano A (2009) A comparative study of axis I antecedents before age 18 of unipolar depression, bipolar disorder and schizophrenia. Psychopathology 42:325-332. https://doi.org/10.1159/000232975

Schultze-Lutter F, Debbané M, Theodoridou A, Wood SJ, Raballo A, Michel C, Schmidt SJ, Kindler J, Ruhrmann S, Uhlhaas PJ (2016) Revisiting the Basic Symptom Concept: Toward Translating Risk Symptoms for Psychosis into Neurobiological Targets. Front Psychiatry 7:9. https://doi.org/10.3389/fpsyt.2016.00009

Schultze-Lutter F, Koch E (2010). Schizophrenia Proneness Instrument, Child and Youth version (SPI-CY). Roma, Giovanni Fioriti Editore s.r.l.

Schultze-Lutter F, Michel C, Schmidt SJ, Schimmelmann BG, Maric NP, Salokangas RK, Riecher-Rössler A, van der Gaag M, Nordentoft M, Raballo A, Meneghelli A, Marshall M, Morrison A, Ruhrmann S, Klosterkötter J (2015) EPA guidance on the early detection of clinical high risk states of psychoses. Eur Psychiatry 30:405-416. https://doi.org/10.1016/j.eurpsy.2015.01.010

Yung AR, Phillips LJ, McGorry PD, McFarlane CA, Francey S, Harrigan S, Patton GC, Jackson HJ (1998) Prediction of psychosis. A step towards indicated prevention of schizophrenia. Br J Psychiatry Suppl 172:14-20

## sTable 1: Overview of life events included in the MEL and their short form used in this paper

| **Domain** | **Item No.** | **Short form of reported LEs** | **Life event (LE)** |
| --- | --- | --- | --- |
| Education | 1 (s) | School-started | Apprenticeship/school started/resumed |
|  | 2 (s) | School-completed | Apprenticeship/school completed resp. final examination passed |
|  | 3 (s) | School-failed | Final examination failed/apprenticeship discontinued |
|  | 4 (s) | School-partner completed | Patient’s partner has completed important apprenticeship or passed final examination |
|  | 5 (s) |  | Partner has failed final examination/discontinued apprenticeship |
|  | 6 (open) |  | Other, not yet mentioned, but very important educational event |
| occupation and household | 7 (l.a.) |  | Lost your job / been dismissed |
|  | 8 (s) (l.a.) | Work-resigned | Gave up job at own request or resigned from job |
|  | 9 (s) (l.a.) | Work-new job | Started new job |
|  | 10 (l.a.) |  | Resumed your professional activity after a break of at least 3 years |
|  | 11 (n.a.) |  | Retired (pension) |
|  | 12 (l.a.) |  | Has moved up the career ladder/significant professional success |
|  | 13 (l.a.) |  | Has lost job or has had very serious professional failure |
|  | 14 (s) | Work-new tasks | Completely new activity/task area at the workplace |
|  | 15 (s) | Work-changed conditions | Significant change in working conditions due to change of classmates/teachers (or colleagues/supervisors) |
|  | 16 (c) | Work-unemployed | Been unemployed for 3 months or more |
|  | 17 (c) | Work-sick-leave | Unable to work/attend school (e.g. due to illness) for at least 3 months |
|  | 18 (c) |  | Performed military/civilian service |
|  | 19 (c) | Work-disputes | At work/school tensions/disputes with colleagues/superiors/teachers for at least 3 months |
|  | 20 (c) | Work-overload | Overload due to school/vocational/homemaker activities for at least 3 months |
|  | 21 (c) | Work-satisfying | great satisfaction about school/vocational/homemaker activities for at least 3 months |
|  | 22 (open) |  | Other, not yet mentioned, but very important occupational event |
| occupation  (main earner) | 23(s) | Main earner-promotion | Main earner has moved up the career ladder/significant professional success |
|  | 24 (s) | Main earner-dismissal | Main earner has lost his job or has had very serious professional failure |
|  | 25 (c) | Main earner-unemployed | Main earner is unemployed foe 3 months or more |
|  | 26 (open) |  | Other, not yet mentioned, occupational event of the main earner with significant impact on patient’s life |
| marriage and love/partner relationship | 27 (s) | Love-started | Love relationship of at least 3 months duration started |
|  | 28 (s) | Love-move in together | Moved into an apartment together with your partner |
|  | 29 (s) | Love-ended | Love relationship ended after a duration of 3 months or more |
|  | 30 (s) |  | Got married |
|  | 31 (s) |  | Git divorced |
|  | 32 (c) | Love-local distance | Lived separately from your partner for at least 3 months (e.g. for professional reasons or because of moving away with parents) |
|  | 33 (c) |  | Lived separately from your partner for at least 3 months because of partnership problems |
|  | 34 (s) |  | Moved back in with partner after a separation of at least 3 months |
|  | 35 (s) |  | Learned about partner’s cheating |
|  | 36 (c) | Love-no partner | No partner relationship for a at least 3 months |
|  | 37 (c) | Love-no sex | Suffering from lack of sexual contact for 3 months or more |
|  | 38 (c) | Love-disputes | Severe tensions/disputes in partner relationship for 3 months or more |
|  | 39 (c) | Love-satisfying | Very satisfied with partner relationship for 3 months or more |
|  | 40 (open) |  | Other, not yet mentioned, but very important love relationship event |

| **Domain** | **Item No.** | **Short form of reported LEs** | **Life event (LE)** |
| --- | --- | --- | --- |
| pregnancy/children | 41 (s) |  | Learned about pregnancy, own or partner’s |
|  | 42 (s) |  | Birth of a healthy child |
|  | 43 (s) |  | Birth of a child with disabilities |
|  | 44 (s) |  | Still birth |
|  | 45 (s) |  | Abortion |
|  | 46 (n.a.) |  | Child(ren) moved out of parent’s home |
|  | 47 (c) |  | Separation of at least 1 year from child(ren) |
|  | 48 (c) |  | Severe tensions/disputes with child(ren) for 3 months or more |
|  | 49 (c) |  | Exceptionally good relationship with child(ren) for 3 months or more |
|  | 50 (open) |  | Other, not yet mentioned, but very important parenting event |
| parents/family | 51(s) | Parents-moved out | Moved out of the parental home |
|  | 52 (c) | Parents-dispute | Severe tensions/disputes with parents for 3 months or more |
|  | 53 (c) | Parents-satisfying | Exceptionally good relationship with parents for 3 months or more |
|  | 54(s) | Parents-moved back in | Moved back into the parental home after living elsewhere for 1 year or more |
|  | 55 (c) | Relatives-dispute | Severe tensions/disputes with cohabiting relatives (not parents) for 3 months or more |
|  | 56 (open) |  | Other, not yet mentioned, but very important family event |
| social contacts/leisure activities | 57 (s) | Friendship-started | Beginning of a longer lasting (at least 3 months) friendship |
|  | 58 (s) | Friendship-ended | End of a longer lasting (at least 3 months) friendship |
|  | 59 (c) | Friendship-none | No close friend/confident for 3 months or more |
|  | 60 (c) | Leisure-restrictions | Severe restriction of leisure activities |
|  | 61 (c) | Leisure-increases | Strong increase in leisure activities |
|  | 62 (c) | Friendship-stressful | Stressed due to disputes among friends for 3 months or more |
|  | 63 (c) | Friendship-satisfying | Extremely satisfying contact with friends for 3 months or more |
|  | 64 (open) |  | Other, not yet mentioned, but very important friendship event |
| deaths of close relatives | 65 (s) |  | Death of spouse |
|  | 66 (s) |  | Death of child |
|  | 67 (s) | Death-parent | Death of a parent |
|  | 68 (s) | Death-friend/relative | Death of a close friend/relative |
| housing | 69 (s) | Housing-relocation | Relocation/change of residence |
|  | 70 (n.a.) |  | Major renovation / construction of the house |
|  | 71 (c) | Housing-poor condition | Extremely poor housing conditions for at least one year |
|  | 72 (open) |  | Other, not yet mentioned, but very important housing event |
| finances | 73 (s) | Income-increased | Significant improvement of the financial situation |
|  | 74( s) | Income-deteriorated | Significant deterioration in financial situation (e.g. due to career change, giving up work, financial downgrading, business failures) |
|  | 75 (c) | Income-too low | Serious financial difficulties (e.g. low income, repayment of debts) for 3 months or more |
|  | 76 (open) |  | Other, not yet mentioned, but very important financial event |
| court/  law viola-tion | 77(s) | Court-sentencing | High financial fine or imprisonment for violation of the law |
|  | 78 (c) | Court-proceedings | Involved in court proceedings for 3 months or more |
|  | 79 (open) |  | Other, not yet mentioned, but very important juridical event |
| health/illness | 80 (c) | Health-hospital | Admission to hospital due to a very serious physical / mental illness or an accident |
|  | 81 (c) | Relative-hospital | Close relative was admitted to hospital due to a very serious physical / mental illness or an accident |
|  | 82 (c) | Health-treatment | Medical treatment for a very serious physical/mental illness for 3 months or more |
|  | 83 (c) | Relative-treatment | Close relative under medical treatment for a very serious physical/mental illness for 3 months or more |
|  | 84 (c) | Health-stressed | Heavy time demands due to the care or nursing of a close relative |
|  | 85 (open) |  | Other, not yet mentioned, but very important health event |

(s) = single LE; (c) = chronic LE, mostly defined by a duration of 3 months / 1 year or more; (n.a.) = not applicable in the age-range of the sample; (l.a.) = limited applicability, i.e., not applicable when still in school education or vocational training never started

## sText 2: Subjective and attenuated negative symptoms assessed with the SPI-CY and SIPS, respectively

**Subjective negative symptoms** in terms of the basic symptom concept [Schultze-Lutter, 2020] were assessed by the Adynamia section of the Schizophrenia Proneness Instrument, Child & Youth version (SPI-CY; [Schultze-Lutter & Koch, 2010]). Basic symptoms are subtle, subclinical and subjective disturbances in all dimensions of mental processes such as drive, affect, stress tolerance, thinking, speech, sensory perception, body perception, motor action and central-vegetative functions that are self-experienced with immediate and full insight into their abnormal nature [Schultze-Lutter, 2009; Schultze-Lutter, et al. 2012]. Of basic symptoms, only a subgroup of cognitive and perceptive basic symptoms are considered psychosis-specific and were employed in the two basic symptom criteria [Schultze-Lutter et al., 2012]. If environmental and personal conditions are favorable (e.g., if there is a supportive social network, and the person possesses good social, problem solving and coping skills), basic symptoms can well be coped with as long as their number and/or severity do not overstrain personal resources. Thus, in early phases, the emerging disorder will only become apparent to others, when inadequate coping strategies are employed or compensatory abilities are exhausted and, consequently, BS start to interfere with behavior. As a result, others might observe patients’ self-initiated coping strategies (avoidance strategies, compensatory behaviors and/or self-medication) and emotional reactions (such as feelings of helplessness, anxiety, tension and/or confusion) in response to basic symptoms, e.g., as primary or secondary negative or affective symptoms [Schultze-Lutter, 2020]. That is to say, any basic symptoms might become apparent as functional deficits or negative symptoms (e.g., avolition, attentional impairment, alogia, anhedonia incl. asociality, and affective flattening), as disorganized communication and behavior and/or as other affective changes. For example, social withdrawal presenting as asociality and/or avolition might directly result from a significantly decreased ability to tolerate social interactions, or from a loss of energy or of drive, or might simply be a result of a decrease in the wish for social contacts that is acted upon. Yet, asociality in terms of a secondary negative symptom might as well result from inadequate coping with disturbances of receptive or expressive speech, or a decrease in thought initiative that might result in feeling incompetent in social communication and, consequently, in avoiding them [Schultze-Lutter, 2020].

In the SPI-CY, the dimension ‘Adynamia’ mainly constitutes of basic symptoms that phenomenologically resemble observable negative symptoms but are primarily self-observed [Schultze-Lutter, 2020]:

- exhaustibility, reduced energy, drive, and persistence, impaired tolerance to certain stressors and lack of purposive thoughts (that may be regarded as the subjective counterpart of observable avolition);
- a general decrease in emotional responsiveness, a decrease in positive emotional responsiveness, and disturbances in presenting oneself (that may be regarded as the subjective counterpart of observable affective flattening);
- concentration disturbances, scatterbrainedness and slowed-down thinking (that may be regarded as the subjective counterpart of observable attentional impairment).

The single basic symptoms included in Adynamia are rated for severity in terms of their frequency of occurrence on a scale ranging from 0=absent to 6=daily and persistent. Additionally, a rating of 7 can be applied, if the symptom has always been present in the same severity, thus not meeting the general basic symptom requirement of being experienced as a change from the ‘normal’ self; a rating of 8 can be applied, if the basic symptom is definitely present but its frequency cannot be established; And a rating of 9 can be applied, if a basic symptom can neither be ruled in nor ruled out. In summary measures of Adynamia, scores of 7 and 9 enter as 0, a score of 8 enters as 1 [Schultze-Lutter & Koch, 2010].

**Attenuated negative symptoms** were assessed by the Negative Symptoms section of the Structured Interview for Psychosis-Risk Syndromes (SIPS; [McGlashan et al., 2010]). In the SIPS, ratings are based to different degrees on information from several sources – patient reports, interviewer observations, third person reports and medical records. The Negative Symptoms include the following six items that are rated syndromally based on interviewer observations and patient reports along a dimension ranging from 0=absent to 6=extreme:

- Social Anhedonia, ranging from being slightly socially awkward (=1) to having no friends and preferring to be alone (=6);
- Avolition, ranging from having less focus on goal-directed activities than considered average (=1) to lack of participation in any goal-directed activities (=6);
- Expression of emotions, ranging from a slightly delayed or delayed emotional responsiveness (=1) to a flat affect, monotone speech, noninvolvement with the interviewer or in the interview (=6);
- Experience of emotions and self, ranging from muted feeling and feeling distant from others (=1) to feeling profoundly changed and possibly alien to self or a loss of feelings (=6);
- Ideational richness, ranging from some conversational awkwardness (=1) to inability to follow even a simple conversation or to give more than single-word responses (=6);
- Occupational functioning, ranging from maintenance of the usual level of work/school performance requiring more than average focus and effort (=1) to failing/leaving employment/school (=6).

In summary measures, the raw scores of the six items were used without further pre-processing.

**References of sText 2**:

McGlashan TH, Walsh B, Woods SW (2010). The psychosis-risk syndrome. Handbook for diagnosis and follow-up. New York: Oxford University

Schultze-Lutter F (2009). Subjective symptoms of schizophrenia in research and the clinic: the basic symptom concept. Schizophr Bull. 35:5-8. https://doi.org/10.1093/schbul/sbn139

Schultze-Lutter F (2020). Basic symptoms in deficit states and their relation to negative symptoms. In: Bitter I (ed.). Managing Negative Symptoms in Schizophrenia. Oxford, UK; Oxford University Press: pp. 19-38. https://doi.org/10.1093/med/9780198840121.003.0002

Schultze-Lutter F, Koch E (2010). Schizophrenia Proneness Instrument, Child and Youth version (SPI-CY). Roma, Giovanni Fioriti Editore s.r.l.

Schultze-Lutter F, Ruhrmann S, Fusar-Poli P, Bechdolf A, Schimmelmann BG, Klosterkötter J (2012). Basic symptoms and the prediction of first-episode psychosis. Curr Pharm Des. 18(4):351-357. https://doi.org/10.2174/138161212799316064.

## sTable 2: Frequency and subjective appraisals of five-year-LEs (all groups n=112)

| **LE No.** | **LE (short form)** | **Frequency of any 1 five-year-LEs**  % (⮉ 1.96<standardized residual<-1.96 ⮋) | | | | **Subjective evaluation (very positive=1 – very negative=5) of most stressful or latest chronic LE**  mean±SD, median (Kruskal-Wallis-H test) | | | | | **Subjective burden (not stressful=1 – extremely stressful=5) of most stressful or latest chronic LE**  mean±SD, median (Kruskal-Wallis-H test) | | | | |
| --- | --- | --- | --- | --- | --- | --- | --- | --- | --- | --- | --- | --- | --- | --- | --- |
|  |  | **CHR-P** | **ClinS** | **GPS** | **χ²(2), *p*** | **CHR-P (I)** | **ClinS (II)** | **GPS (III)** | **H(2), *p*** | **Post-hoc/ H(1), *p*** | **CHR-P (I)** | **ClinS (II)** | **GPS (III)** | **H(2), *p*** | **Post-hoc/ H(1), *p*** |
| 1 | School-started | 47.3% | 37.5% | 57.1% | 8.685, .013 | 2.3±1.1, 2 | 2.5±1.1, 2 | 1.7±0.9, 1 | 16.733, <.001 | I>III, II>III | 2.5±1.1, 2 | 2.6±1.2, 3 | 1.9±1.1, 2 | 13.713, .001 | I>III, II>III |
| 2 | School-completed | 15.2% | 9.8% ⮋ | 37.5% ⮉ | 29.269, <.001 | 2.1±1.2, 2 | 1.5±0.7, 1 | 2.1±1.2, 2 | 2.812, .245 |  | 2.9±1.3, 3 | 1.6±1.0, 1 | 1.7±0.8, 1.5 | 14.500, <.001 | I>II, I>III |
| 3 | School-failed | 4.5% | 2.7% | 2.7% | 0.752, .798 | 4.4±0.6, 4 | 2.7±1.2, 2 | 3.7±0.6, 4 | 5.188, .075 |  | 4.2±0.8, 4 | 2.7±1.5, 3 | 2.3±2.3, 2 | 5.283, .071 |  |
| 4 | School-partner completed | 0.9% | 0.0% | 0.0% | 2.006, 0.367 | 1.0±a, 1 |  |  | b |  | 1.0±a, 1 |  |  | b |  |
| 8 | Work-resigned | 0.9% | 0.0% | 0.9% | 1.006, .605 | 3.0±a, 3 |  | 5.0±a, 5 |  | 1.000, .317 | 4.0±a, 4 |  | 5.0±a, 5 |  | 1.000, .317 |
| 9 | Work-new job | 0.9% | 0.0% | 2.7% | 3.542, .170 | 3.0±a, 3 |  | 2.7±0.6, 3 |  | 0.333, .564 | 3.0±a, 3 |  | 1.7±0.6, 2 |  | 2.000, .157 |
| 14 | Work-new tasks | 8.0% | 7.1% | 5.4% | 0.653, .721 | 2.4±0.7, 3 | 2.5±0.9, 2.5 | 1.5±0.6, 1.5 | 6.003, .049 |  | 2.8±0.8, 3 | 2.9±1.1, 3 | 2.0±0.6, 2 | 3.962, .138 |  |
| 15 | Work-changed conditions | 19.6% | 17.0% | 17.0% | 0.365, .833 | 3.7±1.1, 4 | 3.5±1.2, 4 | 3.0±1.5, 3 | 2.352, .309 |  | 2.8±1.4, 3 | 2.8±1.3, 3 | 2.2±1.3, 2 | 2.991, .224 |  |
| 16 | Work-unemployed | 0.9% | 0.0% | 0.0% | 2.006, 0.367 | 5.0±a, 5 |  |  | b |  | 5.0±a, 5 |  |  | b |  |
| 17 | Work-sick-leave | 8.9% | 25.0% ⮉ | 2.7% ⮋ | 27.725, <.001 | 3.2±1.6, 3.5 | 3.8±1.1, 4 | 3.7±0.6, 4 | 0.985, .611 |  | 3.2±1.7, 3 | 3.5±1.3, 3 | 4.3±0.6, 4 | 1.447, .485 |  |
| 19 | Work-disputes | 40.2% | 33.9% | 18.8% ⮋ | 12.728, .002 | 4.3±0.9, 4 | 4.4±0.6, 4.5 | 4.3±0.6, 4 | 0.644, .725 |  | 4.3±1.0, 5 | 4.1±1.1, 4 | 4.0±1.1, 4 | 1.151, .562 |  |
| 20 | Work-overload | 33.0% ⮉ | 22.3% | 7.1% ⮋ | 22.989, <.001 | 4.2±0.5, 4 | 4.2±0.6, 4 | 3.1±1.1, 3 | 9.165, .010 | I>III, II>III | 4.1±0.9, 4 | 4.1±0.9, 4 | 3.5±0.9, 3.5 | 3.372, .185 |  |
| 21 | Work-satisfying | 18.8% | 14.3% | 8.9% | 4.502, .105 | 1.8±1.0, 2 | 1.6±0.5, 2 | 1.0±0.0, 1 | 8,872, .012 | I>III, II>III | 1.6±1.3, 1 | 1.4±0.5, 1 | 1.1±0.3, 1 | 1.989, .370 |  |
| 23 | Main earner-promotion | 6.3% | 8.0% | 8.9% | 0.584, .747 | 2.0±1.0, 2 | 2.0±0.9, 2 | 2.4±0.7, 2.5 | 1.270, .530 |  | 1.7±1.1, 1 | 1.4±0.7, 1 | 1.1±0.3, 1 | 2.648, .266 |  |
| 24 | Main earner-dismissal | 6.3% ⮉ | 1.8% | 0.9% | 6.390, .041 | 3.0±0.6, 3 | 4.0±0.0, 4 | 4.0±a, 4 | 5.143, .076 |  | 2.1±0.9, 2 | 2.0±0.0, 2 | 4.0±a, 4 | 2.777, .249 |  |
| **LE No.** | **LE (short form)** | **CHR-P** | **ClinS** | **GPS** | **χ²(2), *p*** | **CHR-P (I)** | **ClinS (II)** | **GPS (III)** | **H(2), *p*** | **Post-hoc/ H(1), *p*** | **CHR-P (I)** | **ClinS (II)** | **GPS (III)** | **H(2), *p*** | **Post-hoc/ H(1), *p*** |
| 25 | Main earner-unemployed | 10.7% | 8.9% | 4.5% | 3.141, .208 | 3.4±1.2, 3.5 | 3.4±1.1, 3.5 | 3.0±1.4, 4 | 0.185, .912 |  | 2.5±1.8, 1.5 | 2.1±0.9, 2 | 2.0±1.0, 2 | 0.079, .961 |  |
| 27 | Love- started | 34.8% | 21.4% | 22.3% | 6.497, .039 | 1.4±0.7, 1 | 1.2±0.4, 1 | 1.2±0.4, 1 | 1.936, .380 |  | 1.6±0.9, 1.5 | 1.6±1.1, 1 | 1.4±0.7, 1 | 0.973, .615 |  |
| 28 | Love-move in together | 0.0% | 0.0% | 0.9% | 2.006, 0.367 |  |  | 4.0±a, 4 | b |  |  |  | 4.0±a, 4 | b |  |
| 29 | Love- ended | 27.7% ⮉ | 16.1% | 11.6% | 10.245, .006 | 3.5±1.1, 3 | 3.4±1.2, 4 | 3.2±1.4, 3 | 0.369, .832 |  | 3.2±1.3, 3 | 2.8±1.4, 3 | 2.6±1.1, 3 | 3.206, .201 |  |
| 32 | Love-local distance | 0.0% | 0.0% | 0.9% | 2.006, 0.367 |  |  | 3.0±a, 3 | b |  |  |  | 4.0±a, 4 | b |  |
| 36 | Love-no partner | 17.0% ⮉ | 5.4% | 5.4% | 12.011, .002 | 2.7±1.2, 3 | 3.5±1.1, 3.5 | 2.0±0.9, 2 | 5.420, .067 |  | 1.8±1.3, 1 | 2.3±1.0, 2 | 1.2±0.4, 1 | 4.926, .085 |  |
| 37 | Love-no sex | 0.9% | 0.0% | 0.0% | 2.006, 0.367 | 4.0±a, 4 |  |  | b |  | 3.0±a, 3 |  |  | b |  |
| 38 | Love-disputes | 2.7% | 2.7% | 0.9% | 1.167, .558 | 4.3±0.6, 4 | 4.3±0.6, 4 | 4.0±a, 4 | 0.400, .819 |  | 4.3±0.6, 4 | 4.3±0.6, 4 | 4.0±a, 4 | 0.400, .819 |  |
| 39 | Love-satisfying | 9.8% | 4.5% | 4.5% | 3.657, .161 | 1.4±0.5, 1 | 1.0±0.0, 1 | 1.4±0.6, 1 | 2.521, .283 |  | 1.2±0.4, 1 | 1.0±0.0, 1 | 1.2±0.5, 1 | 1.051, .591 |  |
| 51 | Parents-moved out | 8.0% ⮉ | 1.8% | 1.8% | 7.842, .020 | 3.1±1.7, 3 | 3.0±2.8, 3 | 4.0±1.4, 4 | 0.674, .714 |  | 3.6±1.1, 4 | 2.5±2.1, 2.5 | 3.5±2.1, 3.5 | 0.834, .659 |  |
| 52 | Parents-dispute | 33.9% ⮉ | 28.6% | 1.8% ⮋ | 39.455, <.001 | 4.1±0.7, 4 | 4.3±0.8, 4 | 4.0±0.0, 4 | 1.489, .475 |  | 4.0±1.0, 4 | 4.2±1.0, 4.5 | 3.5±2.1, 3.5 | 1.476, .478 |  |
| 53 | Parents-satisfying | 16.1% | 17.9% | 10.7% | 2.444, .295 | 1.2±0.4, 1 | 1.3±0.4, 1 | 1.0±0.0, 1 | 3.445, .179 |  | 1.1±0.3, 1 | 1.0±0.0, 1 | 1.0±0.0, 1 | 3.630, .163 |  |
| 54 | Parents-moved back in | 1.8% | 0.9% | 1.8% | 0.406, .816 | 1.5±0.7, 1.5 | 1.0±a, 1 | 1.5±0.7, 1.5 | 0.667, .717 |  | 1.5±0.7, 1.5 | 1.0±a, 1 | 1.5±0.7, 1.5 | 0.667, .717 |  |
| 55 | Relatives-dispute | 9.8% | 10.7% | 2.7% | 6.086, .048 | 4.3±0.7, 4 | 3.8±0.6, 4 | 4.0±0.0, 4 | 3.920, .141 |  | 3.6±1.4, 4 | 3.3±1.4, 3.5 | 3.7±0.6, 4 | 0.517, .772 |  |
| 57 | Friendship-started | 64.3% | 68.8% | 77.7% | 4.983, .083 | 1.3±0.5, 1 | 1.5±0.7, 1 | 1.1±0.4, 1 | 17.962, <.001 | II>III | 1.2±0.5, 1 | 1.3±0.7, 1 | 1.0±0.3, 1 | 5.090, .078 |  |
| 58 | Friendship-ended | 34.8% | 38.4% | 38.4% | 0.408, .816 | 3.9±0.9, 4 | 3.7±1.0, 4 | 3.9±0.9, 4 | 0.475, .789 |  | 3.7±1.2, 4 | 3.3±1.4, 3 | 2.9±1.3, 3 | 6.855, .032 | I>III |
| 59 | Friendship-none | 11.6% | 17.0% ⮉ | 3.6% ⮋ | 10.640, .005 | 4.0±0.7, 4 | 3.8±0.8, 4 | 4.8±0.5, 5 | 4.766, .092 |  | 3.6±1.0, 4 | 3.1±1.5, 3 | 4.8±0.5, 5 | 5.312, .070 |  |
| 60 | Leisure-restrictions | 20.5% | 26.8% ⮉ | 7.1% ⮋ | 15.183, <.001 | 3.9±0.7, 4 | 3.8±1.0, 4 | 3.1±1.0, 3 | 4.319, .115 |  | 3.0±1.4, 3 | 3.2±1.1, 3 | 1.6±0.7, 1.5 | 10.367, .006 | I>III, II>III |
| **LE No.** | **LE (short form)** | **CHR-P** | **ClinS** | **GPS** | **χ²(2), *p*** | **CHR-P (I)** | **ClinS (II)** | **GPS (III)** | **H(2), *p*** | **Post-hoc/ H(1), *p*** | **CHR-P (I)** | **ClinS (II)** | **GPS (III)** | **H(2), *p*** | **Post-hoc/ H(1), *p*** |
| 61 | Leisure-increases | 11.6% | 16.1% | 11.6% | 1.308, .520 | 2.0±1.3, 2 | 1.4±0.6, 1 | 1.1±0.4, 1 | 5.050, .080 |  | 1.6±1.0, 1 | 1.4±0.9, 1 | 1.4±0.5, 1 | 0.166, .920 |  |
| 62 | Friendship-stressful | 8.0% | 5.4% | 8.0% | 0.808, .668 | 4.2±0.8, 4 | 4.0±0.6, 4 | 3.8±0.4, 4 | 2.044, .360 |  | 3.6±1.4, 4 | 3.5±1.4, 3.5 | 3.4±0.9, 3 | 0.131, .936 |  |
| 63 | Friendship-satisfying | 19.6% | 15.2% | 9.8% | 4.278, .118 | 1.1±0.4, 1 | 1.7±0.7, 2 | 1.0±0.0, 1 | 15.209, <.001 | I<II, II>III | 1.1±0.2, 1 | 1.1±0.5, 1 | 1.0±0.0, 1 | 0.634, .728 |  |
| 67 | Death-parent | 0.9% | 0.9% | 0.9% | 0.000, 1.0 | 5.0±a, 5 | 5.0±a, 5 | 5.0±a, 5 | 0.000, 1.0 |  | 5.0±a, 5 | 5.0±a, 5 | 5.0±a, 5 | 0.000, 1.0 |  |
| 68 | Death-friend/relative | 28.6% | 28.6% | 30.4% | 0.115, .944 | 4.4±0.7, 5 | 4.5±0.6, 5 | 4.7±0.5, 5 | 3.750, .153 |  | 3.9±1.1, 4 | 4.0±1.0, 4 | 4.0±1.2, 5 | 0.518, .772 |  |
| 69 | Housing-relocation | 25.0% | 19.6% | 25.0% | 1.202, .548 | 2.6±1.2, 3 | 3.2±1.5, 3.5 | 2.0±1.3, 2 | 7.788, .020 | II>III | 2.6±1.3, 2.5 | 2.8±1.7, 2 | 1.7±1.1, 1 | 8.015, .018 | I>III |
| 71 | Housing-poor condition | 0.9% | 0.9% | 0.9% | 0.000, 1.0 | 4.0±a, 4 | 5.0±a, 5 | 5.0±a, 5 | 2.000, .368 |  | 3.0±a, 3 | 5.0±a, 5 | 5.0±a, 5 | 2.000, .368 |  |
| 73 | Income-increased | 11.6% | 8.0% | 14.3% | 2.196, .334 | 1.5±0.5, 1 | 1.3±0.5, 1 | 1.4±0.5, 1 | 0.356, .837 |  | 1.1±0.3, 1 | 1.0±0.0, 1 | 1.3±0.6, 1 | 3.968, .138 |  |
| 74 | Income-deteriorated | 2.7% | 2.7% | 3.6% | 0.206, .902 | 3.7±0.6, 4 | 5.0±0.0, 5 | 4.3±1.0, 4.5 | 4.519, .104 |  | 1.7±1.2, 1 | 4.0±1.0, 4 | 2.8±1.7, 2.5 | 3.517, .172 |  |
| 75 | Income-too low | 1.8% | 0.0% | 0.0% | 4.025, .134 | 4.0±0.0, 4 |  |  | b |  | 4.5±0.7, 4.5 |  |  | b |  |
| 77 | Court-sentencing | 1.8% | 0.9% | 1.8% | 0.406, .816 | 3.5±0.7, 3.5 | 4.0±a, 4 | 3.5±0.7, 3.5 | 0.667, .717 |  | 2.0±1.4, 2 | 4.0±a, 4 | 1.0±0.0, 1 | 3.000, .223 |  |
| 78 | Court-proceedings | 0.0% | 0.9% | 0.9% | 1.006, .605 |  | 5.0±a, 5 | 5.0±a, 5 |  | 0.000, 1.0 |  | 1.0±a, 1 | 5.0±a, 5 |  | 1.000, .317 |
| 80 | Health-hospital | 16.1% | 12.5% | 5.4% | 6.646, .036 | 3.9±0.9, 4 | 3.8±1.1, 4 | 3.7±1.0, 4 | 0.168, .919 |  | 3.5±1.4, 4 | 2.9±1.6, 3 | 2.9±1.5, 2 | 2.159, .340 |  |
| 81 | Relative-hospital | 35.7% ⮉ | 24.1% | 10.7% ⮋ | 19.495, <.001 | 4.1±0.8, 4 | 3.9±0.9, 4 | 4.0±1.1, 4 | 1.760, .415 |  | 3.7±1.3, 4 | 3.3±1.4, 3 | 3.6±1.6, 4 | 1.751, .417 |  |
| 82 | Health-treatment | 36.6% | 50.0% ⮉ | 5.4% ⮋ | 55.302, <.001 | 2.8±0.9, 3 | 2.9±1.3, 3 | 3.8±0.4, 4 | 6.338, .042 | I<III | 2.7±1.2, 3 | 2.5±1.3, 3 | 3.8±1.0, 4 | 7.293, .026 | II<III |
| 83 | Relative-treatment | 17.9% | 15.2% | 8.0% | 4.886, .087 | 3.5±0.9, 4 | 3.2±1.3, 4 | 4.1±0.8, 4 | 3.774, .152 |  | 2.5±1.4, 2 | 1.9±1.1, 1 | 3.1±1.3, 3 | 5.522, .063 |  |
| 84 | Health-stressed | 1.8% | 0.9% | 1.8% | 0.406, .816 | 4.0±1.4, 4 | 4.0±a, 4 | 4.5±0.7, 4.5 | 0.389, .823 |  | 4.0±1.4, 4 | 4.0±a, 4 | 4.0±1.4, 4 | 0.000, 1.0 |  |

CHR-P: clinical high-risk for psychosis; ClinS = inpatient controls; GPS = general population/community controls

a = SD is missing because LE only occurred in n=1; b = Kruskal-Wallis test not feasible due to lack of report of LE in two groups

## sTable 3: Stepwise multinomial regression model of five-year-LEs with significant frequency differences (see sTable 2)

|  | **Clinical High-Risk for Psychosis vs. Community Controls** | | | | | | | **Clinical High-Risk for Psychosis vs. Clinical Controls** | | | | | | |
| --- | --- | --- | --- | --- | --- | --- | --- | --- | --- | --- | --- | --- | --- | --- |
|  | **β (SE)** | **SE** | **Wald** (df=1) | **p** | **Exp(β)** | **95% CI of Exp(β)** | | **β (SE)** | **SE** | **Wald** (df=1) | **p** | **Exp(β)** | **95% CI of Exp(B)** | |
|  |  |  |  |  |  | **lower** | **upper** |  |  |  |  |  | **lower** | **upper** |
| Intercept | 0.270 | 0.320 | 0.712 | .399 |  |  |  | -0.084 | 0.282 | 0.089 | .765 |  |  |  |
| School-started | 1.579 | 0.436 | 13.138 | <.001 | 4.851 | 2.065 | 11.393 | -0.369 | 0.453 | 0.662 | .416 | 0.692 | 0.285 | 1.681 |
| Work-sick leave | -0.597 | 0.784 | 0.581 | .446 | 0.550 | 0.118 | 2.557 | 1.319 | 0.440 | 9.000 | .003 | 3.741 | 1.580 | 8.860 |
| Work-overload | -1.899 | 0.535 | 12.594 | >.001 | 0.150 | 0.052 | 0.427 | -0.581 | 0.341 | 2.909 | .088 | 0.559 | 0.287 | 1.091 |
| Love-no partner | -1.451 | 0.658 | 4.865 | .027 | 0.234 | 0.065 | 0.851 | -1.071 | 0.537 | 3.982 | .046 | 0.343 | 0.120 | 0.981 |
| Parents-moved out | -1.654 | 0.993 | 2.773 | .096 | 0.191 | 0.027 | 1.340 | -1.754 | 0.864 | 4.125 | .042 | 0.173 | 0.032 | 0.940 |
| Parents-dispute | -2.542 | 0.776 | 10.741 | .001 | 0.079 | 0.017 | 0.360 | -0.044 | 0.326 | 0.019 | .892 | 0.957 | 0.505 | 1.812 |
| Friendship-started | 1.335 | 0.374 | 12.751 | <.001 | 3.800 | 1.826 | 7.909 | 0.332 | 0.321 | 1.068 | .301 | 1.393 | 0.743 | 2.614 |
| Friendship-none | -1.399 | 0.721 | 3.760 | .053 | 0.247 | 0.060 | 1.015 | 0.642 | 0.439 | 2.138 | .144 | 1.900 | 0.804 | 4.489 |
| Relative-hospital | -1.335 | 0.472 | 7.989 | .055 | 0.263 | 0.104 | 0.664 | -0.011 | 0.332 | 5.953 | .015 | 0.444 | 0.232 | 0.853 |
| Health-treatment | -2.466 | 0.588 | 19.564 | <.001 | 0.085 | 0.028 | 0.253 | 0.494 | 0.314 | 2.475 | .116 | 1.638 | 0.886 | 3.030 |

SE: standard error; CI: confidence interval

R²=.515 (Nagelkerke), χ²(20)=205.504, p<.001, AIC=468.895, BIC=552.871

## sTable 4: Stepwise multinomial regression model of past-year-LEs with significant frequency differences (see Table 2)

|  | **Clinical High-Risk for Psychosis vs. Community Controls** | | | | | | | **Clinical High-Risk for Psychosis vs. Clinical Controls** | | | | | | |
| --- | --- | --- | --- | --- | --- | --- | --- | --- | --- | --- | --- | --- | --- | --- |
|  | **β (SE)** | **SE** | **Wald** (df=1) | **p** | **Exp(β)** | **95% CI of Exp(β)** | | **β (SE)** | **SE** | **Wald** (df=1) | **p** | **Exp(β)** | **95% CI of Exp(β)** | |
|  |  |  |  |  |  | **lower** | **upper** |  |  |  |  |  | **lower** | **upper** |
| Intercept | 1.277 | 0.196 | 42.342 | <.001 |  |  |  | 0.072 | 0.210 | 0.118 | .731 |  |  |  |
| Work-sick leave | -18.954 | 6616.6 | 0.000 | .998 | <0.001 | 0.000 | a | 1.481 | 0.515 | 8.278 | .004 | 4.396 | 1.603 | 12.056 |
| Work-disputes | -1.425 | 0.544 | 6.876 | .009 | 0.240 | 0.083 | 0.698 | -0.428 | 0.365 | 1.377 | .241 | 0.652 | 0.319 | 1.332 |
| Work-overload | -1.977 | 0.687 | 8.283 | .004 | 0.139 | 0.036 | 0.532 | -0.494 | 0.364 | 1.840 | .175 | 0.610 | 0.299 | 1.246 |
| Parents-moved out | -20.435 | 0.000 | a | a | <0.001 | <0.001 | <0.001 | -2.302 | 1.222 | 3.548 | .060 | 0.100 | 0.009 | 1.098 |
| Parents-dispute | -3.147 | 1.065 | 8.730 | .003 | 0.043 | 0.005 | 0.347 | -0.478 | 0.370 | 1.670 | .196 | 0.620 | 0.300 | 1.280 |
| Relatives-dispute | -20.356 | 8410.8 | 0.000 | .998 | <0.001 | 0.000 | a | -0.040 | 0.538 | 0.006 | .941 | 0.961 | 0.335 | 2.759 |
| Friendship-none | -20.047 | 7498.8 | 0.000 | .998 | <0.001 | 0.000 | a | 0.721 | 0.531 | 1.848 | .174 | 2.057 | 0.727 | 5.819 |
| Health-hospital | -20.435 | 8765.8 | 0.000 | .998 | <0.001 | 0.000 | a | -1.456 | 0.591 | 6.080 | .014 | 0.233 | 0.073 | 0.742 |
| Health-treatment | -3.727 | 1.040 | 12.832 | <.001 | 0.024 | 0.003 | 0.185 | 0.367 | 0.306 | 1.436 | .231 | 1.443 | 0.792 | 2.629 |

SE: standard error; CI: confidence interval; a: not calculable due to too low frequency in at least one group

R²=.488 (Nagelkerke), χ²(18)=190.907, p<.001, AIC=304.904, BIC=381.246

## sTable 5: Subjective appraisals of reported past-year-LEs (all groups n=112)

| **LE No.** | **LE (short form)** | **Subjective evaluation (very positive=1 – very negative=5)**  mean±SD, median | **Subjective burden (not stressful=1 – extremely stressful=5)** mean±SD, median |
| --- | --- | --- | --- |

|  |  | **CHR-P (I)** | **ClinS (II)** | **GPS (III)** | **H(2), *p*** | **Post-hoc/ H(1), *p*** | **CHR-P (I)** | **ClinS (II)** | **GPS (III)** | **H(2), *p*** | **Post-hoc/ H(1), *p*** |
| --- | --- | --- | --- | --- | --- | --- | --- | --- | --- | --- | --- |
| 1 | School-started | 1.9±1.1, 2 | 2.5±1.1, 2 | 1.7±1.0, 1 | 5.159, .076 |  | 2.4±0.9, 2 | 2.4±0.9, 2.5 | 1.9±1.2, 1 | 4.129, .127 |  |
| 2 | School-completed | 2.1±1.4, 1.5 | 1.3±0.6, 1 | 2.7±1.6, 2 | 2.477, .290 |  | 3.0±1.3, 3 | 1.3±0.6, 1 | 1.9±1.0, 2 | 5.291, .079 |  |
| 3 | School-failed | 4.3±0.6, 4 | 2.0±a, 2 | 4.0±0.0, 4 | 3.333, .189 |  | 4.0±1.0, 4 | 1.0±a, 1 | 2.5±0.7, 2.5 | 3.738, .154 |  |
| 4 | School-partner completed | 1.0±a, 1 |  |  | b |  | 1.0±a, 1 |  |  | b |  |
| 9 | Work-new job | 3.0±a, 3 |  | 3.0±a, 3 |  | 0.000, 1.0 | 3.0±a, 3 |  | 2.0±a, 2 |  | 1.000, .317 |
| 14 | Work-new tasks | 1.5±0.7, 1.5 | 3.0±0.0, 3 | 1.3±0.6, 1 | 5.639, .060 |  | 3.0±0.0, 3 | 2.3±1.5, 2 | 2.0±0.0, 2 | 2.174, .337 |  |
| 15 | Work-changed conditions | 3.2±1.3, 3.5 | 2.4±1.3, 2 | 3.5±1.8, 4 | 2.086, .352 |  | 2.3±1.3, 2 | 1.9±1.2, 1 | 2.8±1.6, 3 | 1.409, .494 |  |
| 16 | Work-unemployed | 5.0±a, 5 |  |  | b |  | 5.0±a, 5 |  |  | b |  |
| 17 | Work-sick-leave | 3.3±1.5, 3.5 | 3.7±1.2, 4 |  |  | 0.608, .435 | 3.1±1.7, 3 | 3.4±1.2, 3 |  |  | 0.210, .647 |
| 19 | Work-disputes | 4.2±1.0, 4.5 | 4.4±0.76, 5 | 4.3±0.8, 4.5 | 0.435, .805 |  | 4.2±1.1, 5 | 4.2±1.2, 5 | 3.8±1.0, 3.5 | 1.240, .538 |  |
| 20 | Work-overload | 4.3±0.6, 4 | 4.3±0.6, 4 | 3.3±1.2, 4 | 3.022, .221 |  | 4.2±0.8, 4 | 4.0±0.7, 4 | 3.3±1.2, 4 | 2.591, .274 |  |
| 21 | Work-satisfying | 1.9±1.02, 1 | 1.4±0.5, 1 | 1.0±0.0, 1 | 4.596, .100 |  | 1.9±1.6, 1 | 1.1±0.4, 1 | 1.1±0.4, 1 | 1.757, .415 |  |
| 23 | Main earner-promotion | 2.3±1.2, 3 | 1.8±0.8, 2 |  |  | 0.622, .430 | 2.3±1.5, 2 | 1.6±0.9, 1 |  |  | 0.639, .424 |
| 24 | Main earner-dismissal | 3.0±0.7, 3 | 4.0±a, 4 |  |  | 1.600, .206 | 2.2±0.8, 2 | 2.0±a, 2 |  |  | 0.100, .752 |
| 25 | Main earner-unemployed | 3.4±1.2, 3.5 | 3.5±0.6, 3.5 | 2.0±a, 2 | 2.009, .366 |  | 2.1±1.6, 1 | 2.0±0.8, 2 | 1.0±a, 1 | 0.949, .622 |  |
| 27 | Love- started | 1.4±0.7, 1 | 1.3±0.5, 1 | 1.2±0.04, 1 | 1.936, .380 |  | 1.3±0.7, 1 | 1.6±1.1, 1 | 1.3±0.5, 1 | 1.303, .521 |  |
| 29 | Love- ended | 3.6±1.0, 4 | 4.0±1.2, 4 | 3.8±1.1, 4 | 1.042, .594 |  | 3.2±1.2, 3 | 3.7±1.7, 5 | 2.8±0.8, 3 | 1.937, .380 |  |
| 32 | Love-local distance |  |  | 3.0±a, 3 | b |  |  |  | 4.0±a, 4 | b |  |
| 36 | Love-no partner | 2.7±1.2, 3 | 3.8±0.8, 4 | 1.80±0.9, 2 | 8.260, .016 | II>III | 2.0±1.4, 1 | 2.5±0.8, 2 | 1.2±0.5, 1 | 5.639, .060 | II>III |
| 37 | Love-no sex | 4.0±a, 4 |  |  | b |  | 3.0±a, 3 |  |  | b |  |
| 38 | Love-disputes | 4.0±0.0, 4 | 4.3±0.6, 4 |  |  | 0.667, .414 | 4.0±0.0, 4 | 4.3±0.6, 4 |  |  | 0.667, .414 |
| 39 | Love-satisfying | 1.4±0.5, 1 | 1.0±0.0, 1 | 1.3±0.6, 1 | 1.444, .486 |  | 1.2±0.5, 1 | 1.0±0.0, 1 | 1.0±0.0, 1 | 1.200, .549 |  |
| 51 | Parents-moved out | 2.5±1.0, 3 | 5.0±a, 5 |  |  | 2.500, .114 | 3.0±1.2, 3 | 4.0±a, 4 |  |  | 0.667, .414 |
| 52 | Parents-dispute | 4.1±0.7, 4 | 4.4±0.8, 5 | 4.0±a, 4 | 2.609, .271 |  | 4.1±1.0, 4 | 4.3±1.2, 5 | 2.0±a, 2 | 3.433, .180 |  |
| 53 | Parents-satisfying | 1.2±0.5, 1 | 1.3±0.5, 1 | 1.0±0.0, 1 | 3.290, .193 |  | 1.1±0.4, 1 | 1.0±0.0, 1 | 1.0±0.0, 1 | 3.812, .149 |  |
| 54 | Parents-moved back in | 1.05±a, 1 |  | 1.5±0.7, 1.5 |  | 0.500, .480 | 1.0±a, 1 |  | 1.5±0.7, 1.5 |  | 0.500, .480 |
| 55 | Relatives-dispute | 4.23±0.7, 4 | 3.5±0.5, 3.5 |  |  | 4.497, .034 | 3.4±1.5, 4 | 2.9±1.4, 2.5 |  |  | 0.705, .401 |
| 57 | Friendship-started | 1.3±0.5, 1 | 1.3±0.6, 1 | 1.1±0.3, 1 | 6.107, .047 | I>III | 1.2±0.5, 1 | 1.2±0.7, 1 | 1.0±0.0, 1 | 5.387, .068 |  |
| 58 | Friendship-ended | 3.8±1.0, 4 | 3.8±1.2, 4 | 3.5±1.1, 4 | 0.746, .689 |  | 3.8±1.1, 4 | 3.4±1.4, 3 | 2.5±1.5, 2 | 5.929, .052 |  |
| 59 | Friendship-none | 3.9±0.6, 4 | 3.6±0.7, 3.5 |  |  | 0.736, .391 | 3.5±1.2, 4 | 2.8±1.6, 2.5 |  |  | 1.025, .311 |
| 60 | Leisure-restrictions | 3.9±0.5, 4 | 4.0±1.0, 4 | 3.0±1.2, 3 | 3.696, .158 |  | 3.0±1.2, 3 | 3.4±1.0, 3.5 | 1.2±0.5, 1 | 11.382, .003 | 1>III, II>III |
| 61 | Leisure-increases | 2.0±1.4, 1.5 | 1.3±0.5, 1 | 1.2±0.4, 1 | 2.977, .226 |  | 1.6±1.1, 1 | 1.3±0.6, 1 | 1.2±0.4, 1 | 0.882, .643 |  |
| 62 | Friendship-stressful | 4.3±0.8, 4.5 | 4.3±0.6, 4 | 3.5±0.6, 3.5 | 3.330, .189 |  | 3.8±1.2, 4 | 3.3±1.5, 3 | 3.3±1.3, 3 | 0.668, .716 |  |
| 63 | Friendship-satisfying | 1.2±0.4, 1 | 1.8±0.7, 2 | 1.0±0.0, 1 | 11.711, .003 | I<II, II>III | 1.0±0.0, 1 | 1.2±0.6, 1 | 1.0±0.0, 1 | 2.000, .368 |  |
| 67 | Death-parent | 5.0±a, 5 | 5.0±a, 5 |  |  | 0.000, 1.0 | 5.0±a, 5 | 5.0±a, 5 |  |  | 0.000, 1.0 |
| 68 | Death-friend/relative | 4.5±0.7, 5 | 4.6±0.7, 5 | 4.6±0.6, 5 | 0.323, .8513 |  | 4.2±1.0, 4 | 3.9±1.2, 4 | 3.7±1.0, 3.5 | 1.632, .442 |  |
| 69 | Housing-relocation | 2.1±1.2, 2 | 2.6±1.75, 2.5 | 1.0±0.0, 1 | 7.212, .027 | I>III, II>III | 2.1±1.3, 2 | 2.0±1.6, 1 | 1.3±0.7, 1 | 2.934, .231 |  |
| 71 | Housing-poor condition |  | 5.0±a, 5 |  | b |  |  | 5.0±a, 5 |  | b |  |
| 73 | Income-increased | 1.0±0.0, 1 | 5.0±a, 5 |  |  | 2.000, .157 | 4.0±a, 4 | 5.0±a, 5 |  |  | 3.968, .138 |
| 74 | Income-deteriorated | 1.0±0.0, 1 |  |  | b |  | 4.0±0.0, 4 |  |  | b |  |
| 75 | Income-too low |  |  | 3.0±a, 3 | b |  |  |  | 1.0±a, 1 | b |  |
| 77 | Court-sentencing |  | 5.0±a, 5 |  | b |  |  | 1.0±a, 1 |  | b |  |
| 78 | Court-proceedings | 1.0±0.0, 1 | 1.0±0.0, 1 | 1.0±0.0, 1 | 0.000, 1.0 |  | 1.3±0.5, 1 | 1.3±0.5, 1 | 1.2±0.4, 1 | 0.081, .960 |  |
| 80 | Health-hospital | 3.9±0.8, 4 | 3.6±1.3, 3 |  |  | 0.342 .559 | 3.9±1.0, 4 | 2.6±2.2, 1 |  |  | 1.104, .293 |
| 81 | Relative-hospital | 4.1±0.8, 4 | 4.3±0.8, 4 | 3.8±0.5, 4 | 1.778, .411 |  | 3.6±1.2, 4 | 3.2±1.4, 3 | 3.3±1.7, 3.5 | 0.681, .712 |  |
| 82 | Health-treatment | 2.8±0.9, 3 | 2.7±1.3, 3 | 4.0±a, 4 | 1.925, .382 |  | 2.7±1.2, 3 | 2.4±1.32, 2 | 4.0±a, 4 | 3.809, .149 |  |
| 83 | Relative-treatment | 3.5±0.9, 4 | 3.4±1.2, 4 | 3.6±0.6, 4 | 0.007, .996 |  | 2.4±1.3, 2 | 1.9±1.1, 1 | 2.4±1.1, 2 | 1.232, .540 |  |
| 84 | Health-stressed | 5.0±a, 5 |  |  | b |  | 5.0±a, 5 |  |  | b |  |

CHR-P: clinical high-risk for psychosis; ClinS: inpatient controls; GPS: general population/community controls

a = SD is missing because LE only occurred in n=1

b = Kruskal-Wallis test not feasible due to lack of report of LE in two groups

## sTable 6: Correlations of the subjective appraisals of past-year-LEs in the total sample (n=336)

| **LE No.** | **LE (short form)** | **Spearman’s rho** | **p** | **95% CI of rho** | |
| --- | --- | --- | --- | --- | --- |
|  |  |  |  | **lower** | **upper** |
| 1 | School-started | .334 | .020 | .047 | .570 |
| 2 | School-completed | .553 | .008 | .158 | .795 |
| 3 | School-failed | .857 | .029 | .118 | .985 |
| 4 | School-partner completed | a |  |  |  |
| 8 | Work-resigned | a |  |  |  |
| 9 | Work-new job | a |  |  |  |
| 14 | Work-new tasks | -.068 | .874 | -.749 | .683 |
| 15 | Work-changed conditions | .853 | <.001 | .637 | .930 |
| 16 | Work-unemployed | a |  |  |  |
| 17 | Work-sick-leave | .650 | <.001 | .362 | .825 |
| 19 | Work-disputes | .889 | <.001 | .810 | .937 |
| 20 | Work-overload | .499 | <.001 | .252 | .686 |
| 21 | Work-satisfying | .672 | <.001 | .375 | .844 |
| 23 | Main earner-promotion | .473 | .237 | -3.70 | .889 |
| 24 | Main earner-dismissal | .167 | .752 | -.760 | .870 |
| 25 | Main earner-unemployed | .375 | .207 | -.239 | .775 |
| 27 | Love-started | .208 | .222 | -.139 | .510 |
| 28 | Love-move in together | a |  |  |  |
| 29 | Love-ended | .693 | <.001 | .428 | .848 |
| 32 | Love-local distance | a |  |  |  |
| 36 | Love-no partner | .795 | <.001 | .582 | .906 |
| 37 | Love-no sex | a |  |  |  |
| 38 | Love-disputes | 1.000 | 0.000 | b | b |
| 39 | Love-satisfying | .516 | .104 | -.141 | .858 |
| 51 | Parents-moved out | .000 | 1.000 | -.891 | .891 |
| 52 | Parents-dispute | .779 | <.001 | .629 | .874 |
| 53 | Parents-satisfying | .151 | .352 | -1.78 | .449 |
| 54 | Parents-moved back in | 1.000 | 0.000 | - | - |
| 55 | Relatives-dispute | .451 | .069 | -.054 | .774 |
| 57 | Friendship-started | .226 | .008 | .056 | .383 |
| 58 | Friendship-ended | .693 | <.001 | .517 | .813 |
| 59 | Friendship-none | .823 | <.001 | .605 | .925 |
| 60 | Leisure-restrictions | .495 | <.001 | .220 | .697 |
| 61 | Leisure-increases | .646 | <.001 | .349 | .825 |
| 62 | Friendship-stressful | .679 | .011 | .188 | .899 |
| 63 | Friendship-satisfying | .355 | .034 | .020 | .618 |
| 67 | Death-parent | a |  |  |  |
| 68 | Death-friend/relative | .456 | .005 | .140 | .688 |
| 69 | Housing-relocation | .498 | .008 | .134 | .743 |
| 71 | Housing-poor condition | a |  |  |  |
| 73 | Income-increased | 1.000 | 0.000 | b | b |
| 74 | Income-deteriorated | a |  |  |  |
| 75 | Income-too low | a |  |  |  |
| 77 | Court-sentencing | a |  |  |  |
| 78 | Court-proceedings | .391 | .109 | -.108 | .732 |
| 80 | Health-hospital | .403 | .087 | -.077 | .731 |
| 81 | Relative-hospital | .691 | <.001 | .465 | .833 |
| 82 | Health-treatment | .307 | .004 | .096 | .492 |
| 83 | Relative-treatment | .559 | <.001 | .251 | .764 |
| 84 | Health-stressed | a |  |  |  |

CHR-P: clinical high-risk for psychosis; ClinS: inpatient controls; GPS: general population/community controls

a: cannot be calculated because LE present in ≤3 cases

b: cannot be calculated because rho=1.0

## sTable 7: Stepwise multinomial regression model of sum of five-year-LEs and subjective negative symptoms (Adynamia mean score)

|  | **Clinical High-Risk for Psychosis vs. Community Controls** | | | | | | | **Clinical High-Risk for Psychosis vs. Clinical Controls** | | | | | | |
| --- | --- | --- | --- | --- | --- | --- | --- | --- | --- | --- | --- | --- | --- | --- |
|  | **β (SE)** | **SE** | **Wald** (df=1) | **p** | **Exp(β)** | **95% CI of Exp(β)** | | **β (SE)** | **SE** | **Wald** (df=1) | **p** | **Exp(β)** | **95% CI of Exp(β)** | |
|  |  |  |  |  |  | **lower** | **upper** |  |  |  |  |  | **lower** | **upper** |
| Intercept | 2.179 | 0.250 | 76.026 | <.001 |  |  |  | 1.099 | 0.230 | 22.800 | <.001 |  |  |  |
| Adynamia | -4.840 | 0.636 | 57.837 | <.001 | 0.008 | 0.002 | 0.028 | -0.959 | 0.166 | 33.541 | <.001 | 0.383 | 0.277 | 0.530 |

SE: standard error; CI: confidence interval; a: not calculable due to too low frequency in at least one group

R²=.515 (Nagelkerke), χ²(2)=205.584, p<.001, AIC=414.959, BIC=430.227

## sTable 8: Stepwise multinomial regression model of any five-year-LEs and subjective negative symptoms (Adynamia mean score)

|  | **Clinical High-Risk for Psychosis vs. Community Controls** | | | | | | | **Clinical High-Risk for Psychosis vs. Clinical Controls** | | | | | | |
| --- | --- | --- | --- | --- | --- | --- | --- | --- | --- | --- | --- | --- | --- | --- |
|  | **β (SE)** | **SE** | **Wald** (df=1) | **p** | **Exp(β)** | **95% CI of Exp(β)** | | **β (SE)** | **SE** | **Wald** (df=1) | **p** | **Exp(β)** | **95% CI of Exp(β)** | |
|  |  |  |  |  |  | **lower** | **upper** |  |  |  |  |  | **lower** | **upper** |
| Intercept | 2.179 | 0.250 | 76.026 | <.001 |  |  |  | 1.099 | 0.230 | 22.800 | <.001 |  |  |  |
| Adynamia | -4.840 | 0.636 | 57.837 | <.001 | 0.008 | 0.002 | 0.028 | -0.959 | 0.166 | 33.541 | <.001 | 0.383 | 0.277 | 0.530 |

SE: standard error; CI: confidence interval; a: not calculable due to too low frequency in at least one group

R²=.515 (Nagelkerke), χ²(2)=205.584, p<.001, AIC=236.213, BIC=251.482

## sTable 9: Stepwise multinomial regression model of sum of five-year-LEs and attenuated negative symptoms (SIPS-N mean score)

|  | **Clinical High-Risk for Psychosis vs. Community Controls** | | | | | | | **Clinical High-Risk for Psychosis vs. Clinical Controls** | | | | | | |
| --- | --- | --- | --- | --- | --- | --- | --- | --- | --- | --- | --- | --- | --- | --- |
|  | **β (SE)** | **SE** | **Wald** (df=1) | **p** | **Exp(β)** | **95% CI of Exp(β)** | | **β (SE)** | **SE** | **Wald** (df=1) | **p** | **Exp(β)** | **95% CI of Exp(β)** | |
|  |  |  |  |  |  | **lower** | **upper** |  |  |  |  |  | **lower** | **upper** |
| Intercept | 2.177 | 0.256 | 72.555 | <.001 |  |  |  | 1.008 | 0.241 | 17.492 | <.001 |  |  |  |
| SIPS-N | -3.770 | 0.447 | 71.243 | <.001 | 0.023 | 0.010 | 0.055 | -0.823 | 0.164 | 25.258 | <.001 | 0.439 | 0.318 | 0.605 |

SE: standard error; CI: confidence interval; a: not calculable due to too low frequency in at least one group; SIPS-N: attenuated negative symptoms

R²=.481 (Nagelkerke), χ²(2)=187.596, p<.001, AIC=404.618, BIC=419.886

## sTable 10: Stepwise multinomial regression model of any five-year-LEs and attenuated negative symptoms (SIPS-N mean score)

|  | **Clinical High-Risk for Psychosis vs. Community Controls** | | | | | | | **Clinical High-Risk for Psychosis vs. Clinical Controls** | | | | | | |
| --- | --- | --- | --- | --- | --- | --- | --- | --- | --- | --- | --- | --- | --- | --- |
|  | **β (SE)** | **SE** | **Wald** (df=1) | **p** | **Exp(β)** | **95% CI of Exp(β)** | | **β (SE)** | **SE** | **Wald** (df=1) | **p** | **Exp(β)** | **95% CI of Exp(β)** | |
|  |  |  |  |  |  | **lower** | **upper** |  |  |  |  |  | **lower** | **upper** |
| Intercept | 2.177 | 0.256 | 72.555 | <.001 |  |  |  | 1.008 | 0.241 | 17.492 | <.001 |  |  |  |
| SIPS-N | -3.770 | 0.447 | 71.243 | <.001 | 0.023 | 0.010 | 0.055 | -0.823 | 0.164 | 25.258 | <.001 | 0.439 | 0.318 | 0.605 |

SE: standard error; CI: confidence interval; a: not calculable due to too low frequency in at least one group; SIPS-N: attenuated negative symptoms

R²=.481 (Nagelkerke), χ²(2)=187.596, p<.001, AIC=155.146, BIC=170.414

## sTable 11: Stepwise multinomial regression model of any past-year-LEs and subjective negative symptoms (Adynamia mean score)

|  | **Clinical High-Risk for Psychosis vs. Community Controls** | | | | | | | **Clinical High-Risk for Psychosis vs. Clinical Controls** | | | | | | |
| --- | --- | --- | --- | --- | --- | --- | --- | --- | --- | --- | --- | --- | --- | --- |
|  | **β (SE)** | **SE** | **Wald** (df=1) | **p** | **Exp(β)** | **95% CI of Exp(β)** | | **β (SE)** | **SE** | **Wald** (df=1) | **p** | **Exp(β)** | **95% CI of Exp(β)** | |
|  |  |  |  |  |  | **lower** | **upper** |  |  |  |  |  | **lower** | **upper** |
| Intercept | 1.911 | 0.443 | 18.620 | <.001 |  |  |  | -0.759 | 0.666 | 1.299 | .254 |  |  |  |
| Adynamia | -2.340 | 0.885 | 6.987 | .008 | 0.096 | 0.017 | 0.546 | 0.318 | 0.652 | 0.237 | .626 | 1.374 | 0.383 | 4.933 |
| Any past-year-LEs | 0.290 | 0.533 | 0.296 | .586 | 1.337 | 0.470 | 3.801 | 2.141 | 0.714 | 8.986 | .003 | 8.510 | 2.098 | 34.515 |
| Adynamia*Any past-year-LEs | -3.555 | 1.270 | 7.832 | .005 | 0.029 | 0.002 | 0.345 | -1.434 | 0.677 | 4.480 | .034 | 0.238 | 0.063 | 0.899 |

SE: standard error; CI: confidence interval; a: not calculable due to too low frequency in at least one group

R²=.566 (Nagelkerke), χ²(6)=234.839, p<.001, AIC=256.396, BIC=286.933

## sTable 12: Stepwise multinomial regression model of any past-year-LEs and attenuated negative symptoms (SIPS-N mean score)

|  | **Clinical High-Risk for Psychosis vs. Community Controls** | | | | | | | **Clinical High-Risk for Psychosis vs. Clinical Controls** | | | | | | |
| --- | --- | --- | --- | --- | --- | --- | --- | --- | --- | --- | --- | --- | --- | --- |
|  | **β (SE)** | **SE** | **Wald** (df=1) | **p** | **Exp(β)** | **95% CI of Exp(β)** | | **β (SE)** | **SE** | **Wald** (df=1) | **p** | **Exp(β)** | **95% CI of Exp(β)** | |
|  |  |  |  |  |  | **lower** | **upper** |  |  |  |  |  | **lower** | **upper** |
| Intercept | 2.533 | 0.439 | 33.368 | <.001 |  |  |  | 0.103 | 0.507 | 0.042 | .838 |  |  |  |
| SIPS-N | -3.615 | 0,447 | 65.360 | <.001 | 0.027 | 0.011 | 0.065 | -0.879 | 0.168 | 27.247 | <.001 | 0.415 | 0.299 | 0.578 |
| Any past-year-LEs | -0.568 | 0.472 | 1.448 | .229 | 0.567 | 0.225 | 1.429 | 1.063 | 0.526 | 4.092 | .043 | 2.896 | 1.034 | 8.115 |

SE: standard error; CI: confidence interval; a: not calculable due to too low frequency in at least one group

R²=.509 (Nagelkerke), χ²(4)=202.620, p<.001, AIC=174.146, BIC=197.048

## sTable 13: Stepwise multinomial regression model of five-year-LEs with significant frequency differences and subjective negative symptoms (Adynamia mean score)

|  | **Clinical High-Risk for Psychosis vs. Community Controls** | | | | | | | **Clinical High-Risk for Psychosis vs. Clinical Controls** | | | | | | |
| --- | --- | --- | --- | --- | --- | --- | --- | --- | --- | --- | --- | --- | --- | --- |
|  | **β (SE)** | **SE** | **Wald** (df=1) | **p** | **Exp(β)** | **95% CI of Exp(β)** | | **β (SE)** | **SE** | **Wald** (df=1) | **p** | **Exp(β)** | **95% CI of Exp(β)** | |
|  |  |  |  |  |  | **lower** | **upper** |  |  |  |  |  | **lower** | **upper** |
| Intercept | 2.297 | 0.308 | 55.775 | <.001 |  |  |  | 0.788 | 0.272 | 8.394 | .004 |  |  |  |
| School-completed | 1.349 | 0.537 | 6.303 | .012 | 3.855 | 1.344 | 11.055 | -0.614 | 0.470 | 1.710 | .191 | 0.541 | 0.216 | 1.358 |
| Work-sick leave | 0.055 | 0.915 | 0.004 | .952 | 1.056 | 0.176 | 6.346 | 1.587 | 0.488 | 10.677 | .001 | 4.891 | 1.888 | 12.676 |
| Parents-dispute | -1.849 | 0.852 | 4.707 | .030 | 0.157 | 0.030 | 0.836 | 0.215 | 0.353 | 0.370 | .543 | 1.240 | 0.621 | 2.476 |
| Health-treatment | -2.253 | 0.578 | 15.215 | <.001 | 0.105 | 0.034 | 0.326 | 0.500 | 0.319 | 2.461 | .117 | 1.649 | 0.883 | 3.082 |
| Adynamia | -4.754 | 0.741 | 41.157 | <.001 | 0.009 | 0.002 | 0.037 | -1.080 | 0.184 | 34.568 | <.001 | 0.340 | 0.237 | 0.487 |

SE: standard error; CI: confidence interval; a: not calculable due to too low frequency in at least one group

R²=.659 (Nagelkerke), χ²(10)=296.207, p<.001, AIC=440.316, BIC=486.122

## sTable 14: Stepwise multinomial regression model of past-year-LEs with significant frequency differences and subjective negative symptoms (Adynamia mean score)

|  | **Clinical High-Risk for Psychosis vs. Community Controls** | | | | | | | **Clinical High-Risk for Psychosis vs. Clinical Controls** | | | | | | |
| --- | --- | --- | --- | --- | --- | --- | --- | --- | --- | --- | --- | --- | --- | --- |
|  | **β (SE)** | **SE** | **Wald** (df=1) | **p** | **Exp(β)** | **95% CI of Exp(β)** | | **β (SE)** | **SE** | **Wald** (df=1) | **p** | **Exp(β)** | **95% CI of Exp(β)** | |
|  |  |  |  |  |  | **lower** | **upper** |  |  |  |  |  | **lower** | **upper** |
| Intercept | 3.103 | 0.335 | 85.835 | <.001 |  |  |  | 1.184 | 0.293 | 16.369 | <.001 |  |  |  |
| Work-sick leave | -18.597 | 9986.3 | 0.000 | .999 | <0.001 | 0.000 | a | 2.209 | 0.605 | 13.326 | <.001 | 9.111 | 2.782 | 29.836 |
| Main earner-unemployed | -3.141 | 1.294 | 5.889 | .015 | 0.043 | 0.003 | 0.547 | -1.887 | 0.924 | 4.171 | .041 | 0.151 | 0.025 | 0.927 |
| Love-no partner | -1.650 | 0.758 | 4.738 | .030 | 0.192 | 0.043 | 0.848 | -1.209 | 0.597 | 4.103 | .043 | 0.298 | 0.093 | 0.962 |
| Relatives-dispute | -21.794 | 0.000 | a | a | <0.001 | <0.001 | <0.001 | 0.017 | 0.668 | 0.001 | .979 | 1.017 | 0.275 | 3.767 |
| Friendship-none | -19.216 | 0.000 | a | a | <0.001 | <0.001 | <0.001 | 1.229 | 0.633 | 3.777 | .052 | 3.419 | 0.990 | 11.816 |
| Leisure-restrictions | 0.514 | 0.888 | 0.335 | 0.563 | 1.672 | 0.293 | 9.537 | 1.221 | 0.490 | 6.205 | .013 | 3.391 | 1.297 | 8.863 |
| Health-hospital | -21.690 | 0.000 | a | a | <0.001 | <0.001 | <0.001 | -1.991 | 0.690 | 8.330 | .004 | 0.137 | 0.035 | 0.528 |
| Health-treatment | -4.158 | 1.084 | 14.712 | <.001 | 0.008 | 0.002 | 0.131 | 0.085 | 0.344 | 0.061 | .806 | 1.008 | 0.555 | 2.136 |
| Adynamia | -4.858 | 0.699 | 48.304 | <.001 | 0.008 | 0.002 | 0.031 | -1.272 | 0.203 | 39.127 | <.001 | 0.280 | 0.188 | 0.418 |

SE: standard error; CI: confidence interval; a: not calculable due to too low frequency in at least one group

R²=.704 (Nagelkerke), χ²(18)=330.181, p<.001, AIC=366.882, BIC=443.224

## sTable 15: Stepwise multinomial regression model of five-year-LEs with significant frequency differences and attenuated negative symptoms (SIPS-N mean score)

|  | **Clinical High-Risk for Psychosis vs. Community Controls** | | | | | | | **Clinical High-Risk for Psychosis vs. Clinical Controls** | | | | | | |
| --- | --- | --- | --- | --- | --- | --- | --- | --- | --- | --- | --- | --- | --- | --- |
|  | **β (SE)** | **SE** | **Wald** (df=1) | **p** | **Exp(β)** | **95% CI of Exp(β)** | | **β (SE)** | **SE** | **Wald** (df=1) | **p** | **Exp(β)** | **95% CI of Exp(β)** | |
|  |  |  |  |  |  | **lower** | **upper** |  |  |  |  |  | **lower** | **upper** |
| Intercept | 2.450 | 0.357 | 47.105 | <.001 |  |  |  | 0.480 | 0.362 | 1.759 | .185 |  |  |  |
| School-completed | 2.014 | 0.655 | 9.444 | .002 | 7.492 | 2.074 | 27.062 | -0.572 | 0.488 | 1.374 | .241 | 0.565 | 0.217 | 1.468 |
| Work-sick leave | 0.355 | 1.037 | 0.117 | .732 | 1.426 | 0.187 | 10.879 | 1.899 | 0.513 | 13.720 | <.001 | 6.680 | 2.445 | 18.248 |
| Parents-dispute | -6.046 | 1.777 | 11.570 | <.001 | 0.002 | <0.001 | 0.077 | 0.639 | 0.644 | 0.985 | .321 | 1.895 | 0.536 | 6.696 |
| Relatives-dispute | -1.264 | 1.010 | 1.567 | .211 | 0.283 | 0.039 | 2.044 | 0.990 | 0.595 | 2.770 | .096 | 2.692 | 0.839 | 8.639 |
| Friendship-none | -0.575 | 0.922 | 0.389 | .533 | 0.563 | 0.092 | 3.430 | 0.946 | 0.464 | 4.165 | .041 | 2.576 | 1.038 | 6.392 |
| Relative-hospital | -1.149 | 0.598 | 3.684 | .055 | 0.317 | 0.098 | 1.025 | -0.936 | 0.360 | 6.772 | .009 | 0.392 | 0.194 | 0.794 |
| Health-treatment | -0.790 | 0.843 | 0.879 | .348 | 0.454 | 0.087 | 2.368 | 1.444 | 0.576 | 6.285 | .012 | 4.236 | 1.370 | 13.096 |
| SIPS-N | -3.797 | 0.593 | 41.027 | <.001 | 0.022 | 0.007 | 0.072 | -0.712 | 0.255 | 7.791 | .005 | 0.491 | 0.298 | 0.809 |
| Health-treatment*SIPS-N | -11.653 | 6.437 | 3.277 | .070 | <0.001 | <0.001 | 2.621 | -0.647 | 0.399 | 2.625 | .105 | 0.524 | 0.239 | 1.145 |
| Parents-dispute*SIPS-N | 4.615 | 0.938 | 24.213 | <.001 | 101.013 | 16.070 | 634.968 | -0.617 | 0.448 | 1.991 | .169 | 0.540 | 0.224 | 1.300 |

SE: standard error; CI: confidence interval; a: not calculable due to too low frequency in at least one group

R²=.700 (Nagelkerke), χ²(20)=326.972, p<.001, AIC=427.049, BIC=511.025

## sTable 16: Stepwise multinomial regression model of past-year-LEs with significant frequency differences and attenuated negative symptoms (SIPS-N mean score)

|  | **Clinical High-Risk for Psychosis vs. Community Controls** | | | | | | | **Clinical High-Risk for Psychosis vs. Clinical Controls** | | | | | | |
| --- | --- | --- | --- | --- | --- | --- | --- | --- | --- | --- | --- | --- | --- | --- |
|  | **β (SE)** | **SE** | **Wald** (df=1) | **p** | **Exp(β)** | **95% CI of Exp(β)** | | **β (SE)** | **SE** | **Wald** (df=1) | **p** | **Exp(β)** | **95% CI of Exp(β)** | |
|  |  |  |  |  |  | **lower** | **upper** |  |  |  |  |  | **lower** | **upper** |
| Intercept | 2.520 | 0.303 | 69.309 | <.001 |  |  |  | 0.708 | 0.306 | 5.353 | .021 |  |  |  |
| Health-hospital | -20.327 | 0.000 | a | a | <0.001 | <0.001 | <0.001 | -2.026 | 0.705 | 8.258 | .004 | .132 | 0.033 | 0.525 |
| Work-sick leave | -17.612 | 0.000 | a | a | <0.001 | <0.001 | <0.001 | 2.399 | 0.606 | 15.661 | <.001 | 11.007 | 3.356 | 36.104 |
| Main earner-unemployed | -2.911 | 1.212 | 5.767 | .016 | 0.054 | 0.005 | 0.586 | -1.363 | 0.801 | 2.893 | .089 | 0.256 | 0.053 | 1.231 |
| Relatives-dispute | 21.414 | 0.000 | a | a | <0.001 | <0.001 | <0.001 | 0.389 | 0.598 | 0.424 | .515 | 1.476 | 0.457 | 4.763 |
| Friendship-none | -19.377 | 0.000 | a | a | <0.001 | <0.001 | <0.001 | 0.892 | 0.543 | 2.696 | .101 | 2.439 | 0.841 | 7.071 |
| Health-treatment | -2.008 | 1.221 | 2.703 | .100 | 0.134 | 0.012 | 1.471 | 1.491 | 0.616 | 5.851 | .016 | 4.440 | 1.327 | 14.858 |
| SIPS-N | -3.141 | 0.455 | 47.596 | <.001 | 0.043 | 0.018 | 0.106 | -0.790 | 0.215 | 13.466 | <.001 | 0.454 | 0.298 | 0.692 |
| Health-treatment*SIPS-N | -83.753 | 8946.1 | 0.000 | .993 | <0.001 | 0.000 | a | -1.064 | 0.443 | 5.770 | .016 | 0.345 | 0.145 | 0.822 |

SE: standard error; CI: confidence interval; a: not calculable due to too low frequency in at least one group

R²=.657 (Nagelkerke), χ²(16)=294.797, p<.001, AIC=365.568, BIC=434.276


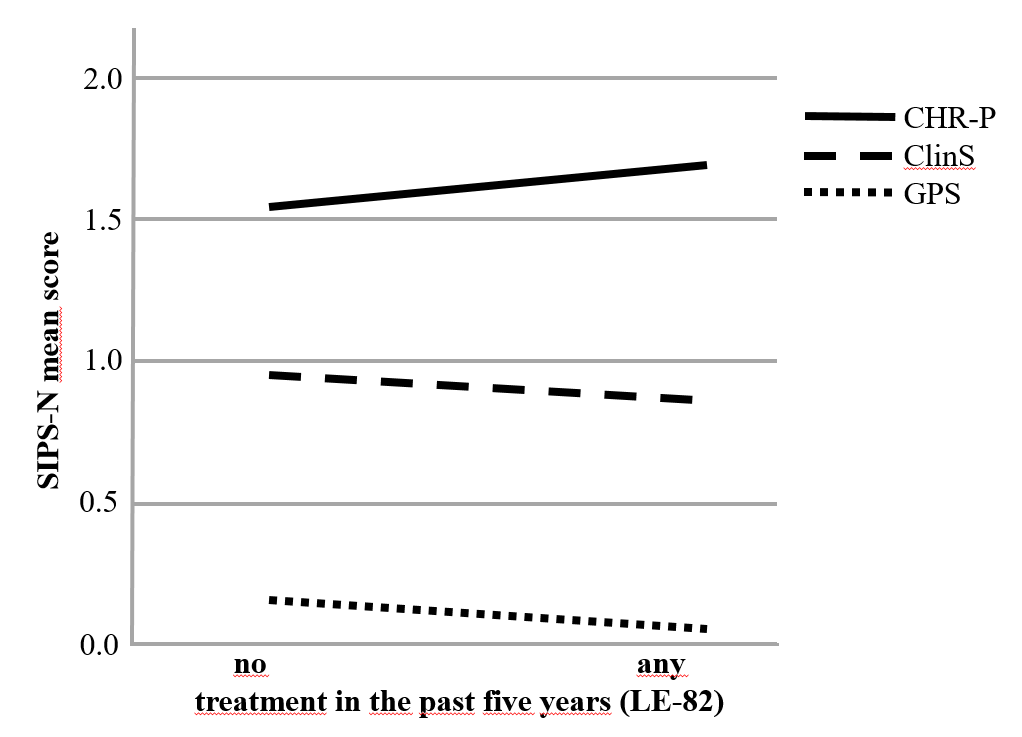


## sFigure 1: Interaction of treatment (LE-82) within the past five years and attenuated negative symptoms (SIPS-N)

CHR-P: clinical high-risk for psychosis; ClinS: inpatient controls; GPS: general population/community controls


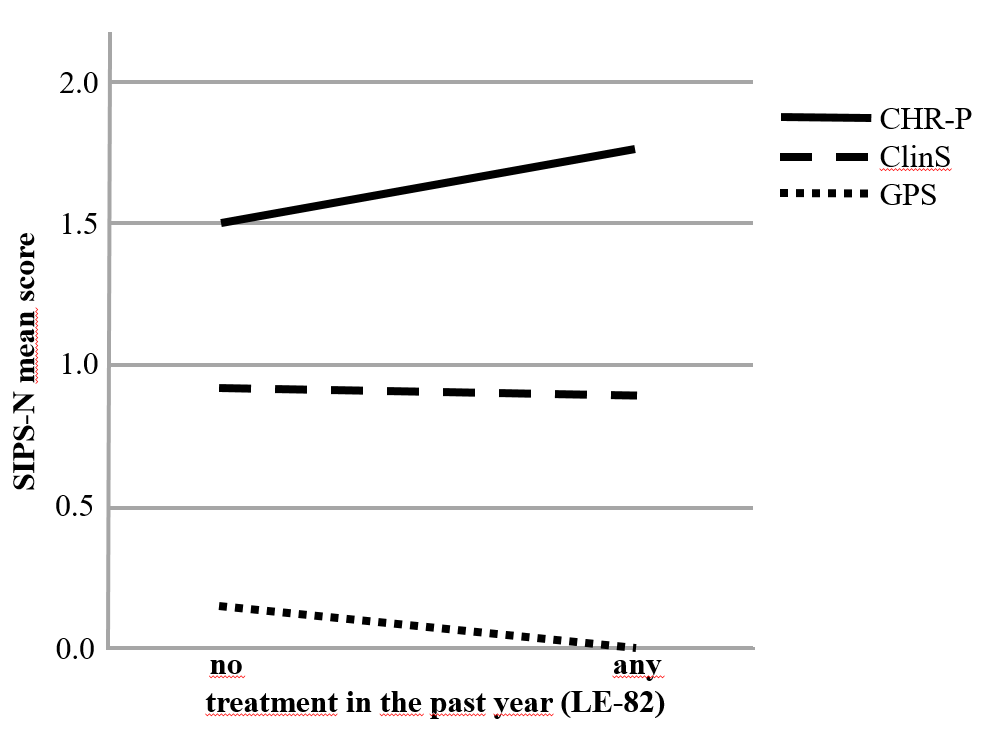


## sFigure 2: Interaction of treatment (LE-82) within the past year and attenuated negative symptoms (SIPS-N)

CHR-P: clinical high-risk for psychosis; ClinS: inpatient controls; GPS: general population/community controls


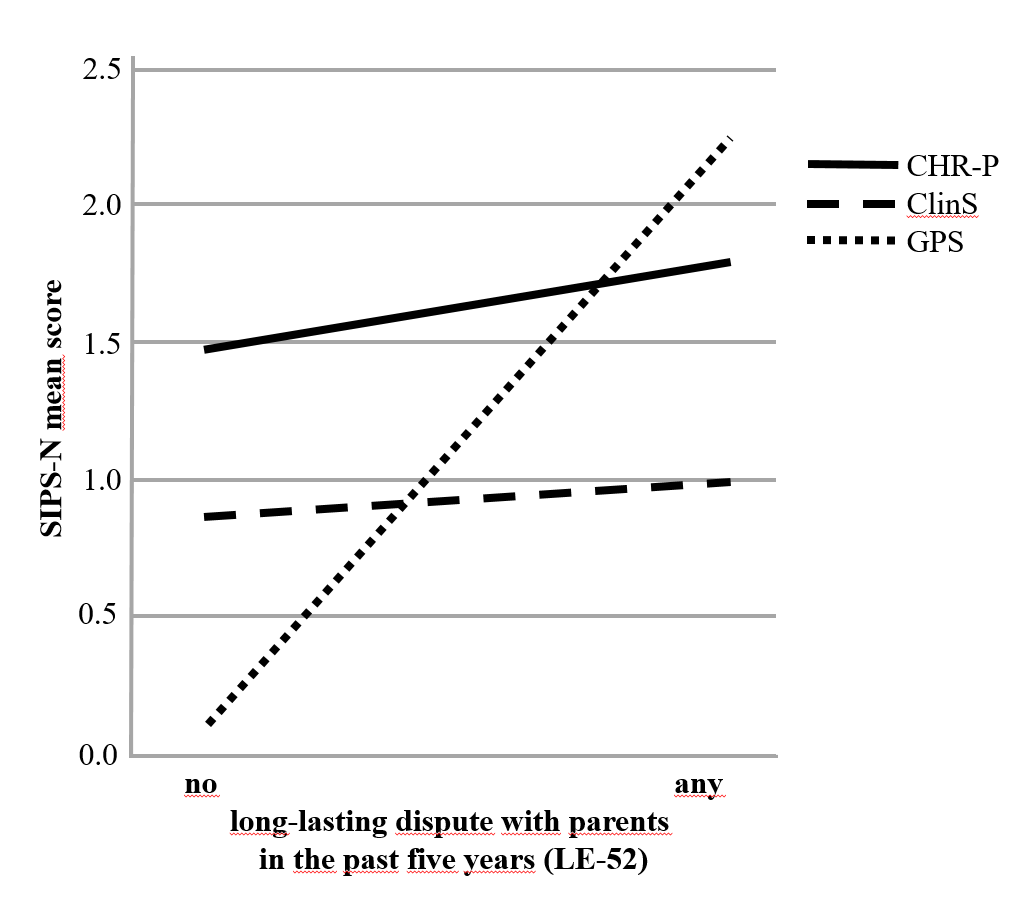


## sFigure 3: Interaction of chronic dispute with parents (LE-52) within the past five years and attenuated negative symptoms (SIPS-N)

CHR-P: clinical high-risk for psychosis; ClinS: inpatient controls; GPS: general population/community controls

## sTable 17: STROBE Statement for cohort studies

|  | Item No | Recommendation | Page No |
| --- | --- | --- | --- |
| **Title and abstract** | 1 | (*a*) Indicate the study’s design with a commonly used term in the title or the abstract | 1 |
|  |  | (*b*) Provide in the abstract an informative and balanced summary of what was done and what was found | 3 |
| Introduction | | | |
| Background/rationale | 2 | Explain the scientific background and rationale for the investigation being reported | 4 |
| Objectives | 3 | State specific objectives, including any prespecified hypotheses | 5 |
| Methods | | | |
| Study design | 4 | Present key elements of study design early in the paper | 5 |
| Setting | 5 | Describe the setting, locations, and relevant dates, including periods of recruitment, exposure, follow-up, and data collection | 5 |
| Participants | 6 | (*a*) Give the eligibility criteria, and the sources and methods of selection of participants. Describe methods of follow-up | 5 |
|  |  | (*b*) For matched studies, give matching criteria and number of exposed and unexposed | 5 |
| Variables | 7 | Clearly define all outcomes, exposures, predictors, potential confounders, and effect modifiers. Give diagnostic criteria, if applicable | 6 |
| Data sources/ measurement | 8* | For each variable of interest, give sources of data and details of methods of assessment (measurement). Describe comparability of assessment methods if there is more than one group | 6 |
| Bias | 9 | Describe any efforts to address potential sources of bias | 5 |
| Study size | 10 | Explain how the study size was arrived at | 5 |
| Quantitative variables | 11 | Explain how quantitative variables were handled in the analyses. If applicable, describe which groupings were chosen and why | 6 |
| Statistical methods | 12 | (*a*) Describe all statistical methods, including those used to control for confounding | 6 |
|  |  | (*b*) Describe any methods used to examine subgroups and interactions | 6 |
|  |  | (*c*) Explain how missing data were addressed | n.a. |
|  |  | (*d*) If applicable, explain how loss to follow-up was addressed | n.a. |
|  |  | (*e*) Describe any sensitivity analyses | n.a. |
| Results | | | |
| Participants | 13* | (a) Report numbers of individuals at each stage of study—eg numbers potentially eligible, examined for eligibility, confirmed eligible, included in the study, completing follow-up, and analysed | 5, sText 1 |
|  |  | (b) Give reasons for non-participation at each stage |  |
|  |  | (c) Consider use of a flow diagram |  |
| Descriptive data | 14* | (a) Give characteristics of study participants (eg demographic, clinical, social) and information on exposures and potential confounders | Table 1 |
|  |  | (b) Indicate number of participants with missing data for each variable of interest |  |
|  |  | (c) Summarise follow-up time (eg, average and total amount) | n.a. |
| Outcome data | 15* | Report numbers of outcome events or summary measures over time | n.a. |
| Main results | 16 | (*a*) Give unadjusted estimates and, if applicable, confounder-adjusted estimates and their precision (eg, 95% confidence interval). Make clear which confounders were adjusted for and why they were included | 6-8 |
|  |  | (*b*) Report category boundaries when continuous variables were categorized | n.a. |
|  |  | (*c*) If relevant, consider translating estimates of relative risk into absolute risk for a meaningful time period | n.a. |
| Other analyses | 17 | Report other analyses done—eg analyses of subgroups and interactions, and sensitivity analyses | 7-8 |
| Discussion | | | |
| Key results | 18 | Summarise key results with reference to study objectives | 8 |
| Limitations | 19 | Discuss limitations of the study, taking into account sources of potential bias or imprecision. Discuss both direction and magnitude of any potential bias | 11 |
| Interpretation | 20 | Give a cautious overall interpretation of results considering objectives, limitations, multiplicity of analyses, results from similar studies, and other relevant evidence | 9-10 |
| Generalisability | 21 | Discuss the generalisability (external validity) of the study results | 11 |
| Other information | | | |
| Funding | 22 | Give the source of funding and the role of the funders for the present study and, if applicable, for the original study on which the present article is based | 2 |

n.a.: not applicable
